# Bio-Based Electrospun Fibers from Chitosan Schiff Base and Polylactide and Their Cu^2+^ and Fe^3+^ Complexes: Preparation and Antibacterial and Anticancer Activities

**DOI:** 10.3390/polym14225002

**Published:** 2022-11-18

**Authors:** Milena Ignatova, Ina Anastasova, Nevena Manolova, Iliya Rashkov, Nadya Markova, Rositsa Kukeva, Radostina Stoyanova, Ani Georgieva, Reneta Toshkova

**Affiliations:** 1 Laboratory of Bioactive Polymers, Institute of Polymers, Bulgarian Academy of Sciences, Acad. G. Bonchev St., Bl. 103A, BG-1113 Sofia, Bulgaria; 2 Institute of Microbiology, Bulgarian Academy of Sciences, Acad. G. Bonchev St., Bl. 26, BG-1113 Sofia, Bulgaria; 3 Institute of General and Inorganic Chemistry, Bulgarian Academy of Sciences, Acad. G. Bonchev St., Bl. 11, BG-1113 Sofia, Bulgaria; 4 Institute of Experimental Morphology, Pathology and Anthropology with Museum, Bulgarian Academy of Sciences, Acad. G. Bonchev St., Bl. 25, BG-1113 Sofia, Bulgaria

**Keywords:** Schiff base, chitosan, electrospinning, polylactide, Cu^2+^ complexes, Fe^3+^ complexes, antibacterial activity, anticancer activity

## Abstract

The Schiff base derivative (Ch-8Q) of chitosan (Ch) and 8-hydroxyquinoline-2-carboxaldehyde (8QCHO) was prepared and fibrous mats were obtained by the electrospinning of Ch-8Q/polylactide (PLA) blend solutions in trifluoroacetic acid (TFA). Complexes of the mats were prepared by immersing them in a solution of CuCl_2_ or FeCl_3_. Electron paramagnetic resonance (EPR) analysis was performed to examine the complexation of Cu^2+^(Fe^3+^) in the Ch-8Q/PLA mats complexes. The morphology of the novel materials and their surface chemical composition were studied by scanning electron microscopy (SEM), attenuated total reflection Fourier transform infrared spectroscopy (ATR-FTIR) and X-ray photoelectron spectroscopy (XPS). The performed microbiological screening demonstrated that in contrast to the neat PLA mats, the Ch-8Q-containing mats and their complexes were able to kill all *S. aureus* bacteria within 3 h of contact. These fibrous materials had efficiency in suppressing the adhesion of pathogenic bacteria *S. aureus*. In addition, Ch-8Q/PLA mats and their complexes exerted good anticancer efficacy in vitro against human cervical HeLa cells and human breast MCF-7 cells. The Ch-8Q-containing fibrous materials had no cytotoxicity against non-cancer BALB/c 3T3 mouse fibroblast cells. These properties render the prepared materials promising as wound dressings as well as for application in local cancer treatment.

## 1. Introduction

Electrospinning is an attractive and low-cost technique for the fabrication of continuous nanoscale polymer fibers. Electrospun fibrous materials exhibit intriguing characteristics such as high surface area-to-volume and aspect ratios and high porosity with very small pore size [1]. Due to these features, the materials are suitable candidates for diverse biomedical applications, such as wound dressings, drug delivery systems, tissue engineering scaffolds, etc. [2,3]. Electrospinning enables the incorporation of drugs of various natures into the polymer fibers. Systems for sustained drug release based on electrospun materials lead to an enhancement in the therapeutic effect of the drugs and a reduction in their side effects [4,5,6]. In recent years bio-based polymers such as the natural polysaccharide chitosan (Ch) and its derivatives, have been considered some of the most promising polymers obtained from renewable sources suitable as drug carriers [7,8,9]. Ch is biocompatible, biodegradable, non-toxic and non-immunogenic; in addition, it bears functional groups that enable its facile chemical modification [10,11]. It also possesses valuable biological properties, among which of particular importance are its haemostatic activity, its ability to influence the function of macrophages, and its good antibacterial, antioxidant and anticancer properties [12,13]. However, Ch is difficult to electrospin due to its limited solubility (in aqueous medium at pH lower than 6 and in harsh solvents) and its polyelectrolyte nature, which makes the necessary entanglement of the chains difficult in many solvents [11,14]. Ch-based fibrous materials have been successfully obtained by electrospinning of Ch alone using TFA as a solvent [15,16,17,18,19] or a mixture of TFA/CH_2_Cl_2_ [15,16,20,21] or in concentrated acetic acid [22,23]. The ability of Ch to be electrospun can be improved by mixing it with water-soluble non-ionogenic polymers with a flexible chain [15,24,25]. In order to improve the mechanical properties of Ch-containing fibrous materials suitable for biomedical applications, the incorporation of aliphatic polyesters into the fibers is of interest. In the present study, poly(L-lactide-*co*-D,L-lactide) (PLA) was selected as the aliphatic polyester because it is a biodegradable polymer with a degradation rate suitable for most musculoskeletal applications. In addition, PLA is a biocompatible polymer of low toxicity and has a good profile of its mechanical properties. These features indicated that PLA-based fibrous materials can serve as suitable candidates for diverse biomedical applications, e.g., implants, drug delivery systems and wound healing materials [26,27,28]. Fibrous materials from Ch or its derivatives and polyesters (e.g., PLA and its copolymers [16,26,29,30,31], poly(ethylene terephthalate) [32,33] and poly(ε-caprolactone) [34]) have been fabricated using TFA as a solvent. Previously, we have reported the preparation of fibrous materials containing Ch or its quaternized derivative by the electrospinning of mixed Ch (quaternized Ch)/PLA solutions in a common solvent TFA/CH_2_Cl_2_ [26]. The prepared materials have been shown to possess high antibacterial activity. We have demonstrated that mats based on quaternized Ch/PLA containing the anticancer drug doxorubicin hydrochloride exert good cytotoxicity in vitro against HeLa, MCF-7 and Graffi tumor cells, and high efficacy in vivo against myeloid Graffi tumor [35,36,37].

8-Hydroxyquinoline and its derivatives are very attractive for biomedical applications due to their beneficial biological properties—antimicrobial, anticancer, antiinflammatory and antioxidant [38,39,40]. In addition, they also possess low toxicity. These compounds have the ability to form complexes with biologically important transition metal ions—Cu^2+^, Fe^2+^, Fe^3+^, etc.—which accounts for their biological action [39,40]. Previously, we have demonstrated that the incorporation of 8-hydroxyquinoline derivatives into electrospun materials from natural [24,41,42,43,44] and synthetic polymers [45,46,47] imparts to them antimicrobial and anticancer activity. We have also shown that 5-amino-8-hydroxyquinoline-modified fibrous materials based on copolymers of styrene and maleic anhydride exhibit high antibacterial and antifungal activity [48]. The possibility of obtaining electrospun materials containing a Schiff base from Jeffamine ED^®^ and 8-hydroxyquinoline-2-carboxaldehyde (8QCHO) or its complex with Cu^2+^, which possess antioxidant and anticancer properties, has also been reported [49]. However, until now, there have been no reports on the preparation of a Schiff base derivative of Ch and 8QCHO (Ch-8Q). An enhancement of the biological activity of the 8-hydroxyquinoline derivatives was observed by their coordination to metal ions of biological significance, such as Cu and Fe ions [38,50]. For this reason, in the present study, we have chosen these ions for the preparation of complexes.

The present study reports the successful fabrication of novel fibrous materials from PLA and Ch-8Q by one-pot electrospinning. Electron paramagnetic resonance (EPR) spectroscopy was used to study the complexation of Cu^2+^ (Fe^3+^) in the complexes of Ch-8Q/PLA mats. The morphology of the mats was examined by scanning electron microscopy (SEM), and their thermal properties were evaluated by differential scanning calorimetry (DSC). The in vitro antibacterial activity of the novel fibrous materials against the Gram-positive bacteria *S. aureus* was assessed. The cytotoxicity of the mats and their complexes against human HeLa and MCF-7 cancer cells as well as non-cancer BALB/c 3T3 mouse fibroblast cells was also studied.

## 2. Materials and Methods

### 2.1. Materials

Poly(L-lactide-*co*-D,L-lactide) (PLA; Resomer^®^ LR 708 (mass average molar mass $\left({\bar{M}}_{w}\right)$ 911,000 g/mol, ${\bar{M}}_{w}$/${\bar{M}}_{n}$ = 2.46; molar ratio L-lactide:D,L-lactide = 69:31) kindly donated by Boerhinger-Ingelheim Chemicals Inc. (Ingelheim am Rhein, Germany) was used in this study. 8-hydroxyquinoline-2-carboxaldehyde (8QCHO) (Aldrich, St. Louis, MO, USA), CuCl_2_ anhydrous (Acros Organics, Geel, Belgium), FeCl_3_ anhydrous (Acros Organics, Geel, Belgium) were used without further purification. Chitosan with an average viscometric molar mass of 380,000 g/mol (Ch) and a degree of deacetylation of 80% was purchased from Aldrich (St. Louis, MO, USA). Glacial acetic acid (Merck, Billerica, MA, USA), absolute ethanol (Merck, Billerica, MA, USA) and trifluoroacetic acid (TFA, Aldrich, St. Louis, MO, USA) of analytical grade purity were used. Dulbecco’s Modified Eagle’s Medium (DMEM) (Sigma-Aldrich, Schnelldorf, Germany), fetal bovine serum (FBS) (Gibso/BRL, Grand Island, NY, USA), glutamine, penicillin and streptomycin (LONZA, Cologne, Germany) were also used in the study. 3-(4,5-Dimethylthiazol-2-yl)-2,5-diphenyltetrazolium bromide (MTT), acridine orange (AO) and ethidium bromide (EtBr) were obtained from Sigma-Aldrich, Schnelldorf, Germany. 4′,6-diamidino-2-phenylindole (DAPI) (AppliChem, Darmstadt, Germany) was used without further purification. The disposable consumables were purchased from Orange Scientific, Braine-l’Alleud, Belgium. Human permanent cancer cells (HeLa, MCF-7) and normal mouse fibroblasts (Balb/c3T3) were purchased from the American Type Culture Collection (ATCC) (Manassas, VA, USA). *Staphylococcus aureus* (*S. aureus*) 3359 were obtained from the National Bank for Industrial Microorganisms and Cell Cultures (NBIMCC), Sofia, Bulgaria.

### 2.2. Preparation of Schiff Base Derivative from Ch and 8QCHO

**Scheme 1:**
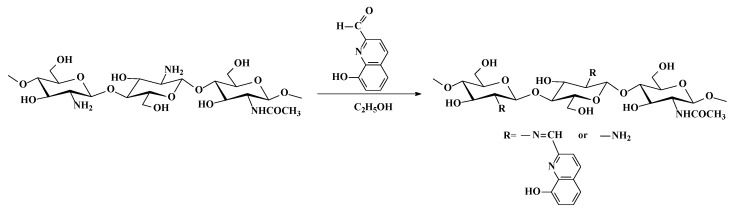
Schematic representation of the synthesis of Ch-8Q.

The synthesis of Ch-8Q is presented in Scheme 1.

To prepare the Schiff base (Ch-8Q) (Scheme 1), Ch (2.5 g, 14.8 mmol) was dissolved in a 125 mL dilute aqueous solution of acetic acid (1% *w*/*v*) for 24 h at 25 °C. Absolute ethanol (13 mL) was added under continuous stirring for 2 h. Then, an ethanolic solution of 8QCHO (2.1 g, 12.1 mmol, 55 mL) was added dropwise to the solution. The mixture was kept under stirring for 24 h at 50 °C. The orange precipitate that formed was filtered and washed with absolute ethanol several times. The unreacted aldehyde was extracted in a Soxhlet apparatus with absolute ethanol for two days. The Schiff base Ch-8Q was dried under reduced pressure at 40 °C for 24 h. Yield—90%. ATR-FTIR (film), ν/cm^−1^: 3410 (ν(N-H), (ν(O-H)), 2886, 2866 (ν(C-H)), 1645 (ν(CH=N)), 1568 (amide II), 1506, 1468 (ν(C=C)), 1373 (δ(C-H)), and 1064 (ν(C-O-C)). The structure of Ch-8Q was also analyzed by ^1^H NMR spectroscopy (Bruker Avance II+ 600, D_2_O/DCl (2%), 333 K). The average degree of substitution was determined from the intensity ratio of the signal at 10.1 ppm for the proton of the imino groups to that at 3.67 ppm for H-2 from the 2-amino-2-deoxy-D-glucopyranose units of Ch. The degree of substitution was 73% for Ch-8Q.

### 2.3. Preparation of Ch-8Q/PLA Fibrous Materials by Electrospinning

Ch-8Q/PLA mixed solutions with Ch-8Q/PLA weight ratio (50:50 and 30:70) were prepared in TFA at a total polymer concentration of 5 wt.%. For comparison, PLA fibers and Ch/PLA fibers (weight ratio of 30:70) were obtained by the electrospinning of their spinning solutions in TFA at a concentration of 5 wt.%.

The system for electrospinning included a plastic syringe equipped with a 22-gauge stainless-steel needle, a syringe infusion pump (NE-300 Just InfusionTM Syringe Pump, New Era Pump Systems Inc., Farmingdale, NY, USA), a high-voltage power supply, and a grounded metal drum collector of diameter 56 mm. The distance between the needle tip and the collector was 13 cm. The applied voltage and flow rates were 30 kV and 1.0 mL/h, respectively. The rotating speed was maintained at 1300 rpm. The humidity was 45% and the temperature was 20 °C. The collected Ch-8Q/PLA fibers were kept under ammonia vapor in a desiccator for 1 h. Then, the mats were dried under reduced pressure at 40 °C for 24 h to remove excess ammonia.

### 2.4. Preparation of the Cu^2+^(Fe^3+^) Complexes of Ch-8Q/PLA Mats, of Ch-8Q, of 8QCHO and of Jeff-8Q

Cu^2+^ or Fe^3+^ complexes of Ch-8Q/PLA mats were obtained by immersing the mats into a 0.1 M absolute ethanol solution of CuCl_2_ or FeCl_3_ for 40 min at 25 °C. The mats were then taken out, purified of non-coordinated salt by washing with absolute ethanol several times and then freeze-dried. For comparison, complexes of Ch/PLA mats were also obtained using this procedure.

Cu^2+^ and Fe^3+^ complexes of Ch-8Q were prepared according to a procedure described in detail in the Supplementary Material (see the preparation of the Cu^2+^(Fe^3+^) complexes of Ch-8Q). Cu^2+^ complexes of 8QCHO and Jeff-8Q were obtained by the procedure described in our previous report [49]. Fe^3+^ complexes of 8QCHO and Jeff-8Q were synthesized by the procedure described in the Supplementary Material (see the preparation of the Fe^3+^ complexes of 8QCHO and Jeff-8Q).

### 2.5. Characterization

Electrospun mats were vacuum-coated with a gold layer using a fine coater Jeol (JFC-1200) and the morphology of the fibrous materials was observed by scanning electron microscopy (SEM, Jeol JSM-5510 (Tokyo, Japan)). The average fiber diameters were calculated over 40 fibers from each SEM image with ImageJ software (V.1.53e, Wayne Rasband, National Institute of Health, Bethesda, MD, USA). Chemical analysis was performed with ATR-FTIR spectroscopy using an IRAffinity-1 spectrophotometer (Shimadzu Co., Kyoto, Japan) equipped with a MIRacleTM ATR (diamond crystal; depth of penetration of the IR beam into the sample was ~2 µm) accessory (PIKE Technologies, Madison, WI, USA). XPS analyses were carried out in the ultrahigh-vacuum (UHV) chamber of an ESCALABMkII (VG Scientific) spectrometer with Mg Kα excitation. DSC analyses were conducted with a DSC TA instrument (DSC Q2000, New Castle, DE, USA) from 0 °C to 400 °C at a 10 °C/min heating rate under nitrogen.

The contact angles were measured by the static sessile drop method using an Easy Drop Krüss GmbH apparatus (DSA 10-MK2 model, Hamburg, Germany) as the average value of at least twenty 10 µL droplets of deionized water placed on the mat surface. The values of the water contact angle were calculated by computer analysis of the acquired images of the droplets.

The EPR spectra of the complexes were acquired as the first derivative of the absorption signal using a Bruker EMXplus EPR spectrometer (E7001039, Karlsruhe, Germany) in the X-band (9.4 GHz). The SpinCount™ software module (Bruker) was used for quantitative EPR calculations. The recorded temperature was changed from 100 to 295 K. The spectra were simulated by the program WIN-EPR SimFonia (Bruker).

### 2.6. Assessment of the Antibacterial Activity

The Ch-8Q/PLA mats and their complexes, PLA and Ch/PLA mats were screened for in vitro antibacterial activity against bacteria *S. aureus* 749 using the viable cell-counting method. Each of the fibrous mats was sterilized by UV irradiation for 30 min and then was added into a suspension of the bacteria (5 mL containing 10^5^ cells/mL). Then the suspension was stirred and incubated at 37 °C. At predetermined time points, 1 mL samples were removed from each tube and serially diluted 10-fold with sterile phosphate-buffered saline (PBS). Finally, the surviving bacteria were counted using the spread-plate method. Triplicate counting was done for each experiment. The number of surviving cells was determined as colony-forming unit (CFU).

The adhesion of bacteria *S. aureus* 749 on the surface of the Ch-8Q/PLA mats and their complexes, and PLA and Ch/PLA mats was assessed using SEM observation with a Jeol JSM-5510 SEM spectrometer (Jeol Ltd., Tokyo, Japan). Mats were incubated in 3 mL of *S. aureus* culture (containing about 10^7^ cells/mL) at 37 °C for 24 h. After incubation, the mats were washed with PBS and then immersed in 2.5 vol.% glutaraldehyde solution in PBS at 4 °C for 5 h. Finally, the mats were washed with PBS, freeze-dried, coated with gold with Jeol JFC-1200 fine coater and studied by a Jeol JSM-5510 SEM spectrometer (Tokyo, Japan).

### 2.7. MTT Cytotoxicity Assay

HeLa cells, MCF-7 cells, or mouse BALB/c 3T3 fibroblasts, were maintained in the logarithmic phase at 37 °C in a humidified atmosphere of 5% CO_2_ in air using Dulbecco’s Modified Eagle Medium (DMEM) (Sigma-Aldrich, Schnelldorf, Germany), containing 10% fetal bovine serum (FBS) (Gibco/BRL, Grand Island, NY, USA), antibiotics (50 units/mL penicillin and 50 µg/mL streptomycin) (LONZA, Cologne, Germany) and 2 mM l-glutamine. Cells were trypsinized by 0.25% Trypsin-EDTA and counted with a hemocytometer. Cell proliferation assay was performed according to the method of Mossman [51]. Briefly, the tested cells (1 × 10^5^ cells/well) seeded in a 96-well microplate were cultivated overnight under standard conditions (37 °C, 5% CO_2_ and 95% humidity) to form a monolayer. After 24 h, the medium was removed and replaced with fresh medium containing different types of fibrous mats (PLA, Ch/PLA, Ch-8Q/PLA, Cu^2+^ complex of Ch-8Q/PLA and Fe^3+^ complex of Ch-8Q/PLA) pre-sterilized with UV irradiation for 30 min. All Ch-8Q-containing mats and their Cu^2+^ and Fe^3+^ complexes were studied at a concentration of 8Q residues 60 µg/mL of culture medium. Cells incubated in culture medium only and in the presence of solutions of Jeff-8Q and its complexes as well as solutions of 8QCHO and its complexes (concentration of 8Q residues was 60 µg/mL of culture medium) were used as negative and positive controls, respectively. Each experimental variant was assayed by five measurements. At the end of 24 h and 72 h incubation, the cells were washed twice with PBS (pH 7.4), and further incubated with 100 μL of MTT solution (Sigma Chemical) at 37 °C for 3 h. Subsequently, the supernatants were aspirated and 100 μL of the lysing solution (DMSO:ethanol = 1:1) was added to each well to dissolve the obtained formazan. The absorbance was measured at 570 nm using a microplate reader (TECAN, Sunrise™, Grodig/Salzburg, Austria). The cell viability was calculated as follows:

(1) $$\mathrm{Cell}\;\mathrm{viability}\;(\%)=\left[\frac{A_{570}(\mathrm{experimental})}{A_{570}\left(\mathrm{control}\right)}\right]\times 100$$

where $A_{570}$(experimental) was the absorbance at 570 nm of the experimental variants and $A_{570}$(control) was the absorbance at 570 nm of the respective negative control.

### 2.8. Fluorescent Microscopic Imaging

#### 2.8.1. Double Staining with AO and EtBr

HeLa cells, MCF-7 cells, or mouse BALB/c 3T3 fibroblasts (1 × 10^5^ cells/well) were cultivated overnight under standard conditions on glass coverslips placed on the bottom of a 24-well tissue culture plate. Following 24 h co-incubation of the cells with the different types of fibrous mats (PLA, Ch/PLA, Ch-8Q/PLA, Cu^2+^ complex of Ch-8Q/PLA and Fe^3+^ complex of Ch-8Q/PLA), the coverslips were rinsed twice with PBS (pH 7.4) and stained with fluorescent dyes—AO (5 µg/mL) and EtBr (5 µg/mL) in the ratio of 1:1—to visualize the cytomorphological alterations. It is known that AO can permeate both live and dead cells and shows strong yellow-green fluorescence after intercalation in DNA. EtBr is a fluorochrome that passes through damaged membranes and shows orange fluorescence as a result of the intercalation in DNA. EtBr stains late apoptotic cells and dead cells.

Cells maintained in the culture medium only, without treatment, served as a negative control. Cells treated with solutions of Jeff-8Q and its Cu^2+^(Fe^3+^) complexes as well as solutions of 8QCHO and its Cu^2+^(Fe^3+^) complexes were used as a positive control. The cells were examined under a fluorescence microscope (Leica DM 5000B; Wetzlar, Germany).

#### 2.8.2. DAPI Staining

The nuclear morphology of the treated and control-untreated cells was further analyzed through DAPI staining according to a standard procedure [52]. For this purpose, the HeLa cells, MCF-7 cells, or mouse BALB/c 3T3 fibroblasts were processed as described in 2.8.1. After 24 h incubation, the glass coverslips were removed and washed with PBS (pH 7.4). Then the cells were fixed with 3% paraformaldehyde at room temperature and stained with a DAPI solution for 15 min at room temperature in the dark. The nuclear morphology of the stained cells was examined under a fluorescence microscope (Leika DM 5000B, Wetzlar, Germany).

### 2.9. Statistical Analysis

Statistical analysis was performed by one-way analysis of variance (ANOVA) followed by Bonferroni’s post hoc comparison test (GraphPad Prism software package, version 5 (GraphPad Sofware Inc., San Diego, CA, USA)). *** *p* < 0.001 was considered statistically significant.

## 3. Results and Discussion

### 3.1. Morphology

The morphology and average diameter of the fibrous materials obtained by electrospinning are affected by various parameters such as the composition of the spinning solution, the concentration of the solution and the applied field strength. For the preparation of fibrous materials from Ch-8Q and PLA, a suitable co-solvent was chosen—TFA—that allowed the preparation of mixed solutions and their successful electrospinning. Defect-free and cylindrical fibers of PLA and Ch/PLA were also obtained.

**Figure 1:**
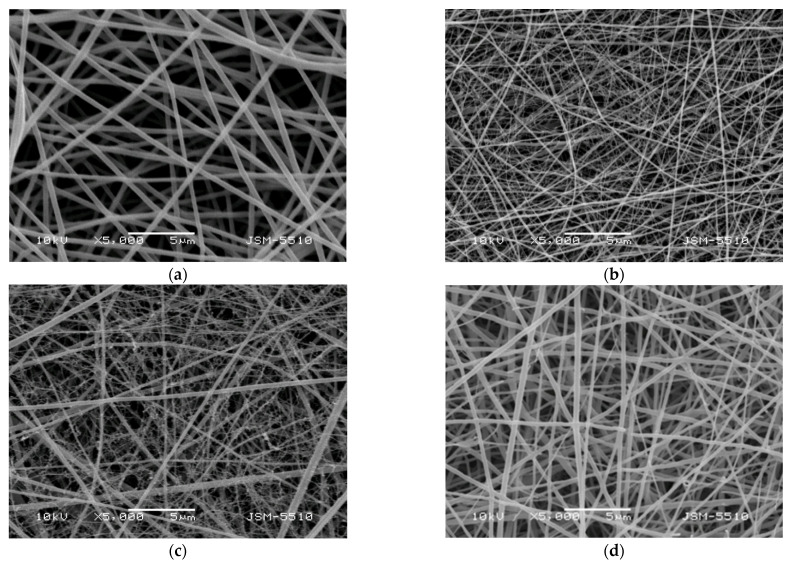
SEM micrographs of electrospun mats of (**a**) PLA, (**b**) Ch-8Q/PLA (50:50 *w*/*w*), (**c**) Ch-8Q/PLA (30:70 *w*/*w*) and (**d**) Ch/PLA (30:70 *w*/*w*); magnification ×5000.

Figure 1 shows the SEM micrographs of the prepared electrospun materials. Fibers prepared from a PLA solution with a concentration of 5 wt.% had an average diameter of 360 ± 90 nm (Figure 1a). A decrease in the average fiber diameter was observed when adding Ch-8Q or Ch to the PLA solution (Figure 1b–d).

For the Ch-8Q/PLA system at a weight ratio of Ch-8Q:PLA = 30:70, the average fiber diameter was 187 ± 128 nm, while at a weight ratio of Ch-8Q:PLA = 50:50, it was 124 ± 45 nm (Figure 1b,c). The average diameter of Ch/PLA fibers (weight ratio Ch:PLA = 30:70) was 238 ± 105 nm. The dynamic viscosities of the solutions of PLA, Ch/PLA (Ch:PLA = 30:70 *w*/*w*), Ch-8Q/PLA (Ch-8Q:PLA = 30:70 *w*/*w*) and Ch-8Q/PLA (Ch-8Q:PLA = 50:50 *w*/*w*) were 4200, 1900, 1700 and 940 cP, respectively. It has been found for other systems that the lower solution viscosity led to a decrease in fiber diameter or an increase in the amount of spindle-like defects [24,43,46,47]. In our study, decreasing the solution viscosity also resulted in the formation of fibers with smaller diameters. Moreover, Ch-8Q/PLA and Ch/PLA fibers are characterized by a broader diameter distribution compared to that of PLA fibers, which explains the elevated standard deviation of the average diameters of the Ch-8Q/PLA and Ch/PLA fibers. Some fiber splitting and branching of the main fibers with the appearance of very thin fibers was observed in the cases of the Ch-8Q/PLA and Ch/PLA mats (Figure 1b–d). The obtained results are consistent with those observed by other authors for Ch and Ch/PLA mats electrospun in TFA [15,29] and are most likely due to the fact that the jet elongation and solvent evaporation alter the shape and charge per unit area of the jet, which may cause some change in the balance between the surface tension and the electrical forces, which may, in turn, allow jet splitting and fiber branching to occur [19,53].

Electrospun mats obtained at a weight ratio of Ch-8Q:PLA = 50:50 proved too brittle to manipulate. Thus, in the present study, mats from Ch-8Q and PLA in a weight ratio of 30:70 were selected for further biological experiments as the optimal formulation.

**Figure 2:**
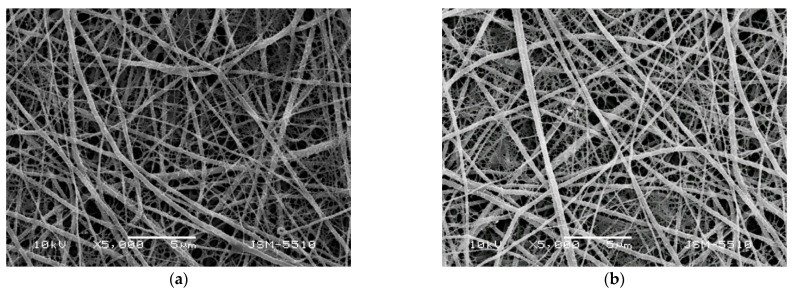
SEM micrographs of electrospun Ch-8Q/PLA (30:70 *w*/*w*) mats after immersion in an ethanol solution of (**a**) CuCl_2_ and (**b**) FeCl_3_ for 40 min.; magnification ×5000.

Experiments were conducted to obtain Cu^2+^ and Fe^3+^ complexes of Ch-8Q/PLA mats by immersing the fibrous materials in an ethanol solution of CuCl_2_ and FeCl_3_ for 40 min. As seen from the SEM micrographs, after this treatment, the mats retained their fibrous structure and the average diameter of the fibers remained unchanged—208 ± 126 nm and 189 ± 130 nm for Cu^2+^ and Fe^3+^ complexes of the Ch-8Q/PLA mats, respectively (Figure 2a,b).

### 3.2. ATR-FTIR Spectra of Fibrous Materials

**Figure 3:**
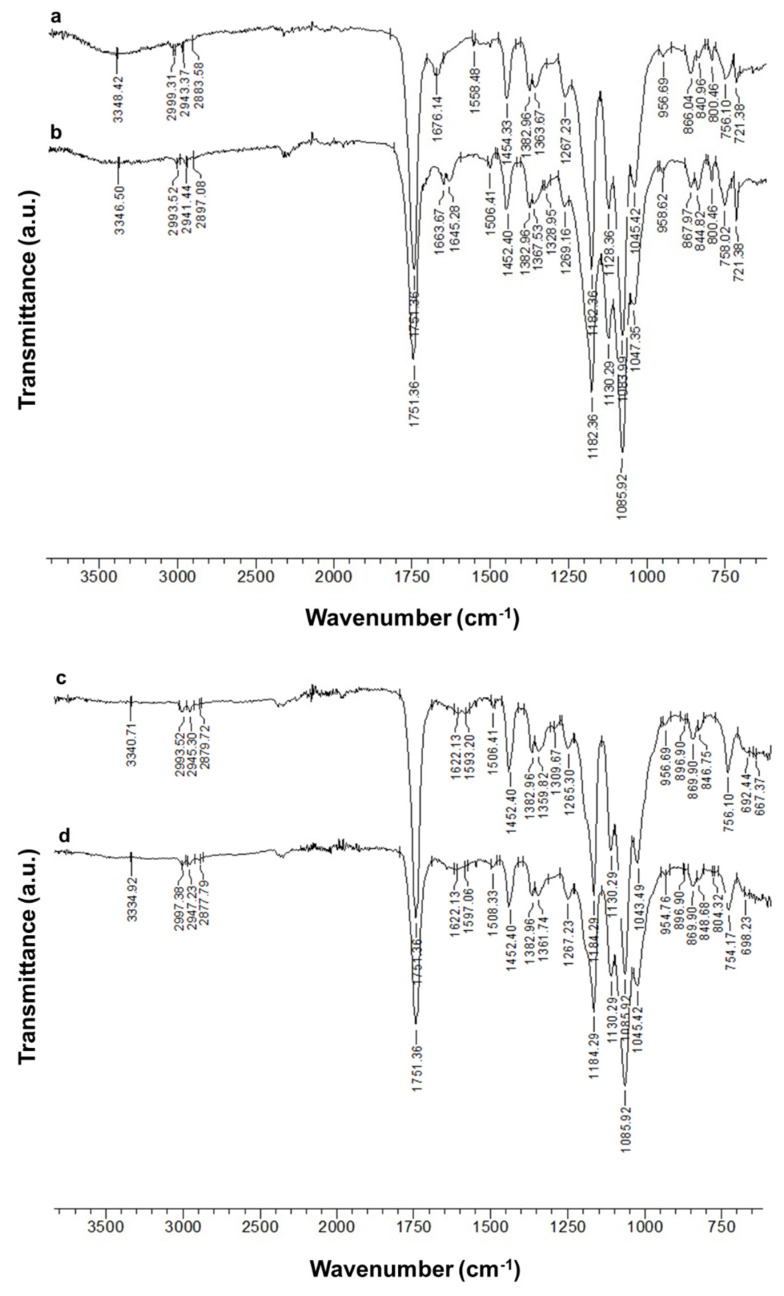
ATR-FTIR spectra of (a) Ch/PLA (70:30 *w*/*w*) mat, (b) Ch-8Q/PLA (70:30 *w*/*w*) mat, (c) Cu^2+^ complex of Ch-8Q/PLA mat and (d) Fe^3+^ complex of Ch-8Q/PLA mat.

ATR-FTIR spectroscopy was used to characterize the Ch-8Q/PLA mats and their Cu^2+^ and Fe^3+^ complexes. In the ATR-FTIR spectrum of the Ch/PLA mats, absorption characteristic bands were detected for both PLA (1751 cm^−1^—C=O stretching vibration; 1084 cm^−1^—C-O stretching vibration) and Ch (1676 cm^−1^— stretching vibrations for protonated amino (-NH_3_^+^) groups; 1558 cm^−1^—amide II; 3348 cm^−1^—O-H and N-H stretching vibrations) (Figure 3 (a)). In the spectrum of the Ch-8Q/PLA mats, in addition to the PLA bands, characteristic bands were recorded at 1664, 1645 and 1506 cm^−1^, respectively, for amide I from the polysaccharide structure of Ch-8Q, for C=N stretching vibrations from the azomethine group of the Schiff base Ch-8Q and for stretching vibrations from the ring of 8Q moieties in Ch-8Q, respectively (Figure 3 (b)). In the ATR-FTIR spectrum of Cu^2+^ and Fe^3+^ complexes of Ch-8Q/PLA mats, a shift of the absorption characteristic band for C=N stretching vibrations from the azomethine group to 1622 cm^−1^, compared to the spectrum of Ch-8Q/PLA mats (1645 cm^−1^) was detected. This shift is most likely due to the coordination of Cu^2+^ and Fe^3+^ to the azomethine nitrogen of Ch-8Q. This is consistent with the literature data for other metal complexes of Schiff bases [54,55]. In addition, in the spectra of the complexes (Figure 3 (c,d)), the band attributed to the C=N stretching vibrations of the 8Q residues in Ch-8Q, which was recorded in the spectrum of 8QCHO at 1591 cm^−1^ (Supplementary Material, Figure S1a), was shifted towards higher wavenumbers by 2 cm^−1^ to 1593 cm^−1^ and by 6 cm^−1^ to 1597 cm^−1^ for Cu^2+^ and Fe^3+^ complexes of the Ch-8Q/PLA mats, respectively. This is most likely due to the fact that the lone pair of electrons on the nitrogen of the 8Q residues participates in bond formation with the metal ion [56].

### 3.3. Thermal Behavior of the Fibrous Mats

**Figure 4:**
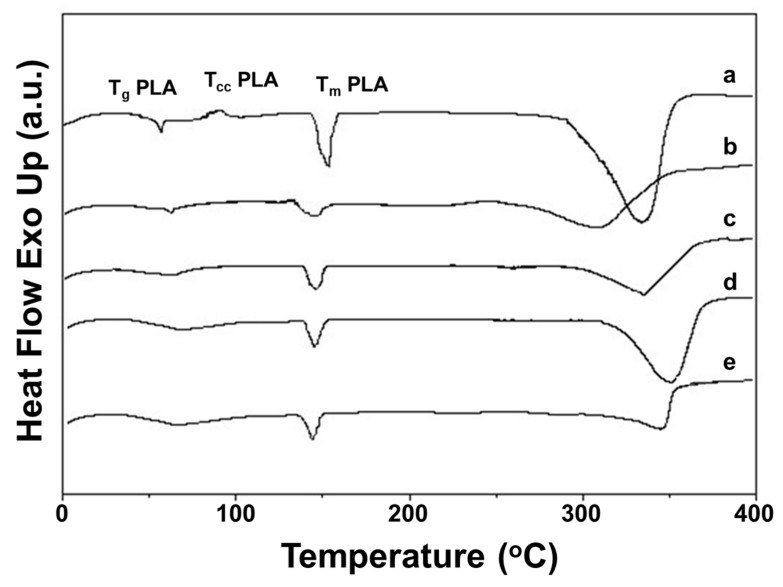
DSC thermograms of mats from (a) PLA, (b) Ch/PLA, (c) Ch-8Q/PLA, (d) Cu^2+^ complex of Ch-8Q/PLA and (e) Fe^3+^ complex of Ch-8Q/PLA.

The thermal properties of Ch-8Q/PLA mats and their Cu^2+^ and Fe^3+^ complexes were studied by DSC (Figure 4). In the DSC thermograms of the Ch-8Q/PLA mats and their complexes and of the Ch/PLA mats, a weakly intense broad endothermic peak between 25 and 100 °C was observed, which might be attributed to desorption of water or TFA from Ch and Ch-8Q (Figure 4 (b–e)). In the thermogram, the glass transition temperature (T_g_), cold crystallization temperature (T_cc_) and melting temperature (T_m_) for the PLA mat were detected at 62 °C, 89 °C and 153 °C, respectively (Figure 4 (a)). The thermograms of Ch/PLA mats, as well as Ch-8Q/PLA and their Cu^2+^ and Fe^3+^ complexes, showed an absence of T_cc_ for PLA (Figure 4 (b–e)). This indicated that mixing with Ch, Ch-8Q and complex formation with the metal ions most likely affects the crystallization and the rate of crystallization of PLA.

Furthermore, the T_g_ for PLA did not change upon the incorporation of Ch and Ch-8Q into the mats, as well as upon coordination of Cu^2^ or Fe^3+^ with the Ch-8Q-containing mats (61 °C for Ch/PLA mats as well as for Ch-N=C-8Q/PLA mats and their complexes) (Figure 4 (b–e)). As seen from Figure 4 (b–e), T_m_ for PLA was observed at a lower temperature—at 148 °C, 147 °C, 145 °C and 145 °C for Ch/PLA, Ch-8Q/PLA mats, and Cu^2+^ and Fe^3+^ complexes of Ch-8Q/PLA mats, respectively. Similarly to Ch, a melting peak for Ch-8Q was not recorded (Figure 4 (b,c)). In the DSC thermograms of the mats at a temperature above 280 °C, the appearance of endothermic peaks was detected (Figure 4 (a–e)), which were most likely due to the thermal degradation of the polymer constituents of the mats. These observations require further profound study to elucidate the changes that occur in the thermal stability of the mats upon the incorporation of Ch-8Q instead of Ch, as well as upon the coordination of Cu^2+^ or Fe^3+^ with the Ch-8Q/PLA mats.

### 3.4. Water Contact Angle Measurements

**Figure 5:**
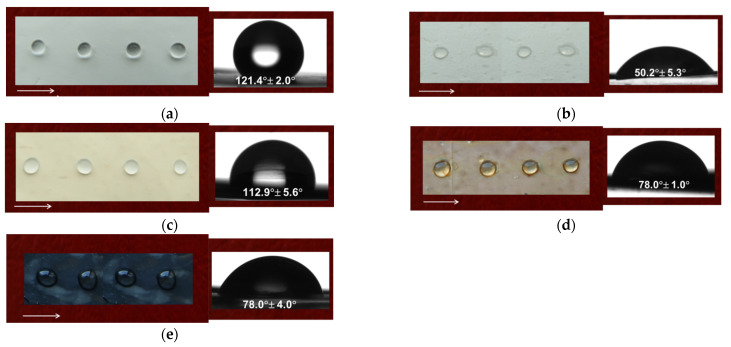
Digital images of water droplets (10 µL) deposited on the surfaces of fibrous mats from (**a**) PLA, (**b**) Ch/PLA, (**c**) Ch-8Q/PLA, (**d**) Cu^2+^ complex of Ch-8Q/PLA and (**e**) Fe^3+^ complex of Ch-8Q/PLA. The direction of the collector rotation is indicated by an arrow.

It is known that the adhesion of cells and their proliferation are highly dependent on the wettability of the surface of electrospun materials [57]. Therefore, in the present study, the wettability of the surface of the obtained fibrous materials that would come in contact with bacterial and cancer cells was measured. The PLA mat had a hydrophobic surface (the value of the water contact angle was 121.4 ± 2.0°) (Figure 5a). The water droplet retained its spherical shape on the surface of the mat. Hydrophilization of the mats was observed when Ch was present in the fibers (Figure 5b). The measured values of the water contact angle for Ch/PLA were 50.2 ± 5.3°. The Ch-8Q/PLA mats were hydrophobic (the water contact angle value was 112.9 ± 5.6°) (Figure 5c). Coordination of Cu^2+^ and Fe^3+^ with the Ch-8Q/PLA mats led to a decrease in the water contact angle to about 78.0° (Figure 5d,e).

### 3.5. XPS Analysis

XPS analysis of the surface also confirmed the structure of the Ch-8Q/PLA mats and their Cu^2+^ and Fe^3+^ complexes. Peaks at 285.0 eV (-C-H or -C-C- of PLA and Ch-8Q, as well as -C-NH_2_ of Ch-8Q), at 285.6 eV (-C-N and -C-OH of the 8Q residues), at 286.8 eV (-C-O, -C-OH and -C-N-C=O of Ch-8Q, -C-O of PLA and -C=N of Ch-8Q), at 288.4 eV (-O-C-O- and -N-C=O of Ch-8Q), at 289.1 eV (-O-C=O of PLA) and at 290.5 eV (π→π* shake-up satellite characteristic of the 8Q ring of Ch-8Q) were detected in the high-resolution C_1s_ spectrum of Ch-8Q/PLA mats (Supplementary Material Figure S2a). The O_1s_ spectrum showed four components at 530.8 eV assigned to -N-C=O of Ch-8Q, at 532.2 eV for -C=O of PLA, at 532.9 eV for -C-OH of Ch-8Q and of the 8Q residues and at 533.5 eV for -O-C-O and -C-O of PLA (Supplementary Material Figure S2b). The N_1s_ signal consisted of four peaks at 398.8 eV, assigned to -N=C of Ch-8Q, at 399.6 eV to -N-C=O and -C-NH_2_ of Ch-8Q, at 400.8 eV characteristic of the –N-C of the 8Q residues in Ch-8Q and at 401.8 eV for the -NH_3_^+^ groups of Ch (Supplementary Material Figure S2c). The theoretically calculated peak-area ratio for the corresponding carbon atoms was [C-C/C-H/C-NH_2_]/[C-N/C-OH]/[C-O/C-OH/C-N-C=O/C-O/C=N]/[O-C-O/N-C=O]/[O-C=O]/[π→π*] = 34.1/2.3/36.1/3.5/23.3/0.7. The experimentally determined ratio was 35.5/2.3/35.3/3.4/22.8/0.7. The peak for the carbon atoms from the C-C/C-H/C-NH_2_ bonds had the largest area. This is consistent with the determined surface hydrophobicity of the Ch-8Q/PLA mat (the water contact angle was 112.9° ± 5.6°).

**Figure 6:**
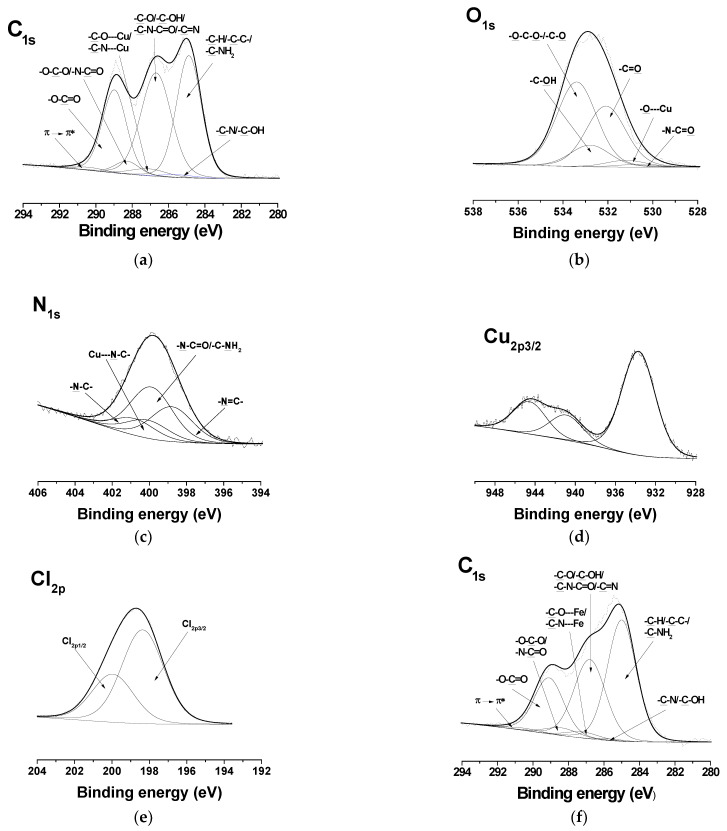

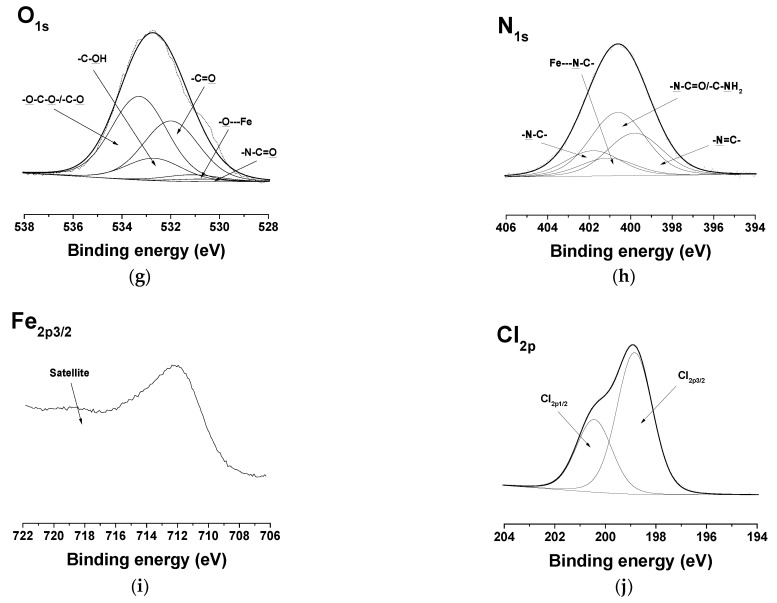
XPS peak fittings for Cu^2+^ complex of Ch-8Q/PLA mat [C_1s_ (**a**), O_1s_ (**b**), N_1s_ (**c**), Cu2p 3/2 (**d**) and Cl2p (**e**)] and Fe^3+^ complex of Ch-8Q/PLA mat [C_1s_ (**f**), O_1s_ (**g**), N_1s_ (**h**), Fe2p 3/2 (**i**) and Cl2p (**j**)].

In the detailed C_1s_ spectra of the complexes of the Ch-8Q/PLA mats, compared with those of the Ch-8Q/PLA mats, the appearance of a new component was observed at 287.2 eV, characteristic of -C-O---Cu/-C-N---Cu or -C-O---Fe/-C-N---Fe (Figure 6a,f). The intensity of the peak at 286.8 eV also decreased. In the expanded O_1s_ spectra of the complexes of the mats, a new -O---Cu or -O---Fe signal from the 8Q residues in Ch-8Q appeared at 531.2 eV (Figure 6b,g). In the N_1s_ spectra of the complexes of the Ch-8Q/PLA mats, a new peak at 400.1 eV was detected, attributed to -C-N---Cu or -C-N---Fe of the 8Q residues in Ch-8Q (Figure 6c,h). The complex formation between Cu^2+^ and the Ch-8Q/PLA mats was also indicated by the appearance of a peak composed of two components—Cu_2p1/2_ and Cu_2p3/2_ (Figure 6d). Cu_2p3/2_ consisted of a main peak at 933.7 eV and two satellites at 940.9 eV and 944.6 eV. The main peak was characterized by a binding energy close to that observed by other authors for Cu^2+^ complexes (933.1 eV) [58]. In the XPS spectrum of the Fe^3+^ complex of the Ch-8Q/PLA mats, a new Fe_2p3/2_ component from the Fe_2p_ region with a main peak at 711.9 eV and a satellite at 718.2 eV was also detected (Figure 6i). The binding energy of the main peak was close to that reported by other authors for Fe^3+^ complexes [59]. These results confirmed the coordination of Cu^2+^ or Fe^3+^ on the surface of the Ch-8Q/PLA mats. The appearance of a peak in the Cl2p region (at 198.4 eV (Cl2p3/2) and 200.0 eV (Cl2p1/2)) (Figure 6e,j) indicated the presence of Cl ions on the surface layer of complexes of the Ch-8Q/PLA mats.

### 3.6. EPR Spectroscopy Analysis of Cu^2+^ and Fe^3+^ Complexes of the Fibrous Materials

**Figure 7:**
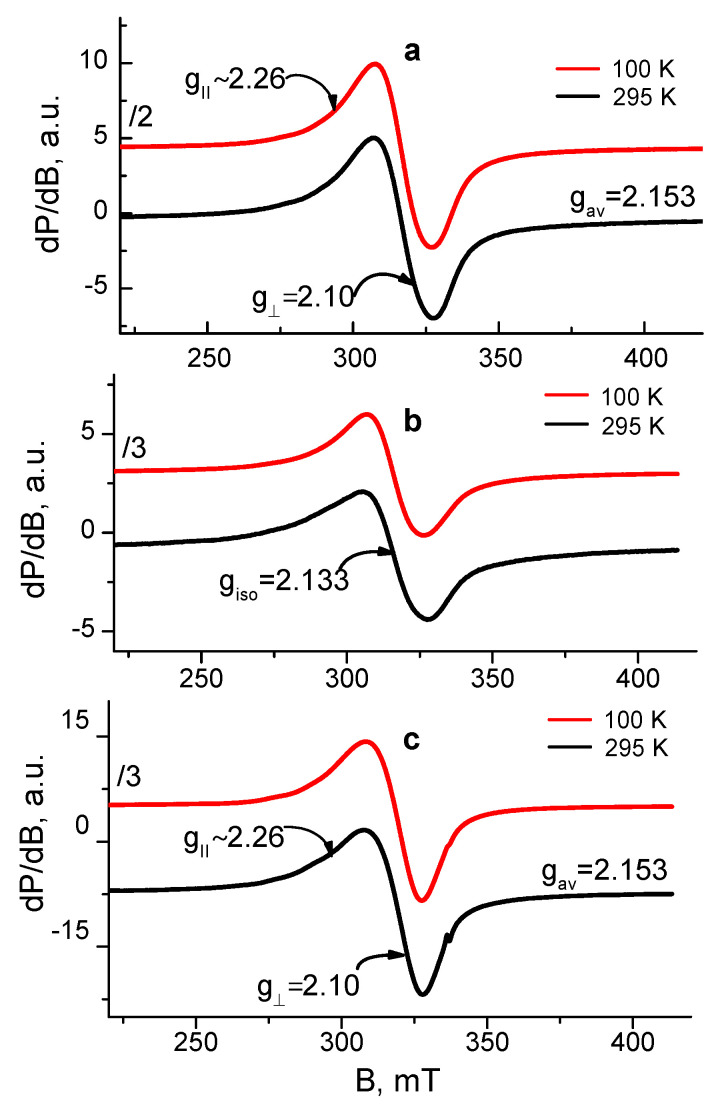
EPR spectra at 100 and 295 K of (**a**) Cu^2+^-Ch-8Q/PLA mat, (**b**) Cu^2+^-Ch/PLA mat and (**c**) Cu^2+^-Ch-8Q in the solid state.

**Figure 8:**
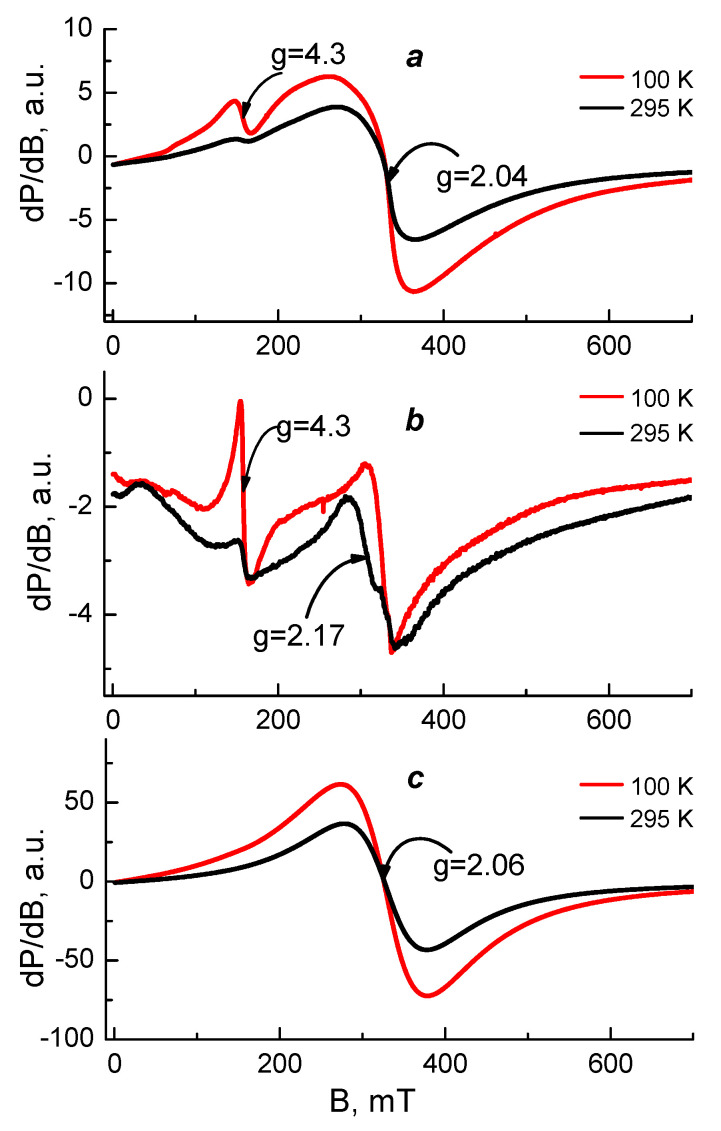
EPR spectra at 100 and 295 K of (**a**) Fe^3+^-Ch-8Q/PLA mat, (**b**) Fe^3+^-Ch/PLA mat and (**c**) Fe^3+^-8QCHO in the solid state.

EPR analysis was used in order to reveal the coordination mechanism of Cu^2+^ and Fe^3+^ in the complexes of Ch-8Q/PLA fibrous materials. As EPR standards, we studied Cu^2+^ and Fe^3+^ complexes of Ch/PLA mats as well as complexes Cu^2+^-Ch-8Q and Fe^3+^-8QCHO in the solid state. The complexes were analyzed in the temperature range of 100 to 295 K and their spectra are shown in Figure 7 and Figure 8.

The EPR spectrum of the Cu^2+^ complex of the Ch-8Q/PLA mats contained one nearly symmetric signal with slightly resolved g_⊥_ and g_∥_ (g_⊥_ = 2.10, g_II_~2.26). The signal retained its shape and position on cooling, while its intensity increased according to the Currie–Weiss law (ϴ = −147 ± 6 K). These EPR parameters are typical for Cu^2+^ ions that are magnetically coupled.

Since the Schiff base Ch-8Q (Scheme 1) used to obtain the Ch-8Q-containing mats is a derivative of Ch and 8QCHO, the possibilities for coordination of Cu^2+^ ions with 8Q residues as well as with Ch moieties could not be excluded. Thus, the question arises as to how the Cu^2+^ ions are coordinated in the complexes of Ch-8Q/PLA mats. To understand the Cu^2+^ coordination, two reference materials were used: Cu^2+^-Ch/PLA mat and Cu^2+^-Ch-8Q in the solid state. The EPR spectrum of the Cu^2+^-Ch/PLA mats complex consisted of one symmetric signal with g = 2.133. The g-value was constant between 100 and 295 K, and the linewidth (∆H_pp_) varied from 22.8 mT at 295 K to 19.7 mT at 100 K. The temperature dependence of the reciprocal value of signal intensity followed the Currie–Weiss law (ϴ = −28 ± 7 K). All these EPR parameters indicated that the EPR signal of the Ch/PLA mats originated from exchanged coupled Cu^2+^ ions. The g-value of the Ch/PLA mats was close to that of g_av_ previously determined by Pawlicka et al. [60] for membranes of Ch coordinated with Cu^2+^ ions (g_av_ = 2.123). Therefore, it could be concluded that Ch is coordinated around the Cu^2+^ ions in Cu^2+^-Ch/PLA mats.

The spectrum of the Cu^2+^-Ch-8Q complex in the solid state showed a slightly asymmetric signal with low-intensity and not fully resolved hyperfine lines. The EPR parameters at 295 K were: g_II_~2.26, g_⊥_ ≈ 2.10, g_av_ = 2.153. The signal asymmetry decreased on cooling from 295 to 100 K, but the temperature dependence of the signal intensity followed the Currie–Weiss law (ϴ = −52 ± 4 K). It is worth noting that the g_iso_ of the Cu^2+^-8Q complex [50] is close to the g_av_ value of the Cu^2+^-Ch-8Q complex. In addition, the EPR parameters of the Cu^2+^-Ch-8Q complex deviated from that of the Cu^2+^-Ch/PLA mat. Therefore, the signal of the solid-state Cu^2+^-Ch-8Q complex was assigned to Cu^2+^ ions that were coordinated by 8Q residues.

The comparison between the EPR parameters of the Cu^2+^-Ch-8Q/PLA mat and the two above-mentioned references allowed the outlining of several EPR features. Because of the difference in the g-value for the Ch/PLA mat and Ch-8Q/PLA mat as well as the close g-value of the Cu^2+^-Ch-8Q/PLA mat and solid Cu^2+^-Ch-8Q complex, it suggested a similar coordination of Cu^2+^ in both materials, i.e., it appeared that Cu^2+^ ions in Ch-8Q/PLA mats are preferentially coordinated with 8Q residues in Ch-8Q. This means that Cu^2+^ ions were coordinated with O and N atoms from the 8Q moieties.

Figure 8 shows the EPR spectra of the studied Fe^3+^ complexes. The EPR spectrum of the Fe^3+^-8QCHO complex consisted of a broad, symmetric signal with g = 2.06 and a linewidth ∆H_pp_ ≈ 100 mT at 295 K. A similar signal with g = 2.04 and ∆H_pp_ ≈ 93 mT at 295 K was observed also in the central part of the Fe^3+^-Ch-8Q/PLA mat spectrum. These signals were assigned to exchanged coupled Fe^3+^ ions and their similarity could be related to similar coordination of Fe^3+^ ions in solid Fe^3+^-8QCHO complex and Fe^3+^-Ch-8Q/PLA mat complex. These findings showed that Fe^3+^ ions in the complex of the Ch-8Q/PLA mat were preferentially coordinated to 8Q residues of Ch-8Q.

In order to confirm this conclusion the EPR spectra of the Fe^3+^-Ch-8Q/PLA mat and Fe^3+^-Ch/PLA mat were compared. Their spectra were characterized by two different signals having g-values of g = 4.3 and g = 2.0. In the EPR spectrum of the Fe^3+^-Ch/PLA mat the signal with g ≈ 4.3 dominated, while the signal with g ≈ 2.0 predominated in the Fe^3+^-Ch-8Q/PLA mat spectrum. Furthermore, the intensity of the signal with g ≈ 2.0 for the Fe^3+^-Ch-8Q/PLA mat was more than 50 times higher than that for Fe^3+^-Ch/PLA mat, thus indicating a higher concentration of coordinated Fe^3+^. Therefore Fe^3+^ was coordinated in Fe^3+^-Ch-8Q/PLA mat preferably by 8Q moieties of Ch-8Q.

### 3.7. Evaluation of the Antibacterial Activity

**Figure 9:**
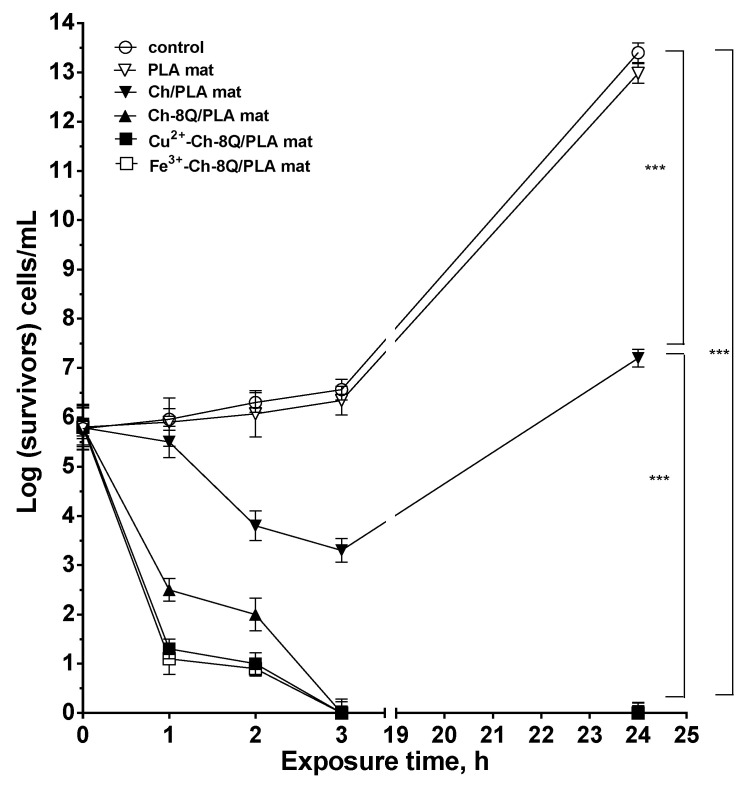
Logarithmic plot of the viable bacterial cell *S. aureus* number versus the exposure time for control (*S. aureus*), PLA mat, Ch/PLA mat, Ch-8Q/PLA mat, Cu^2+^ complex of Ch-8Q/PLA mat and Fe^3+^ complex of Ch-8Q/PLA mat. Data represent the mean ± standard deviation (*n* = 3). *** *p* < 0.001, statistical significance.

The antibacterial activity of the Ch-8Q/PLA mats and their Cu^2+^ and Fe^3+^ complexes against the Gram-positive bacteria *S. aureus* was estimated by counting the viable bacteria that remained after incubation of the mats in an *S. aureus* suspension for given time periods. In the present study, *S. aureus* bacteria were selected because they are one of the most common pathogenic bacteria responsible for secondary infections of wounds. For the sake of comparison, the antibacterial activity of PLA and Ch/PLA mats was also examined. The *S. aureus* control was found to grow normally for the given time periods during the experiment and log(CFU/mL) reached 13.4 in 24 h. As can be seen in Figure 9, the neat PLA mats did not suppress the growth of *S. aureus* bacteria. In this case, the number of survived cells in 24 h was 13.0 log units. In contrast, Ch/PLA mats with a Ch content of 1000 μg/mL, for a contact time of 24 h led to a 7.2 log decrease in the *S. aureus* titer (Figure 9). A difference in the antibacterial activity was observed for the Ch-8Q/PLA mats and their Cu^2+^ and Fe^3+^ complexes at the same content of Ch-8Q—1000 μg/mL. For Ch-8Q/PLA mats, the *S. aureus* titer decreased by 2 log units for a contact time of 2 h, whereas for the same contact time for Cu^2+^ and Fe^3+^ complexes of Ch-8Q/PLA mats, a reduction of the *S. aureus* titer by 1 and 0.9 log units was detected, respectively. In the cases of Ch-8Q/PLA mats and their complexes, viable *S. aureus* cells were absent after 3 h of contact. The obtained results showed that the incorporation of Ch-8Q into the mats, as well as the complexation of the mats with Cu^2+^ and Fe^3+^, imparted to the mats a higher antibacterial activity compared to that of the Ch/PLA mats. The Ch-8Q-containing mats and their complexes that manifest their activity by contact between the bioactive agent and the bacteria are perspective dressings for infected wounds.

**Figure 10:**
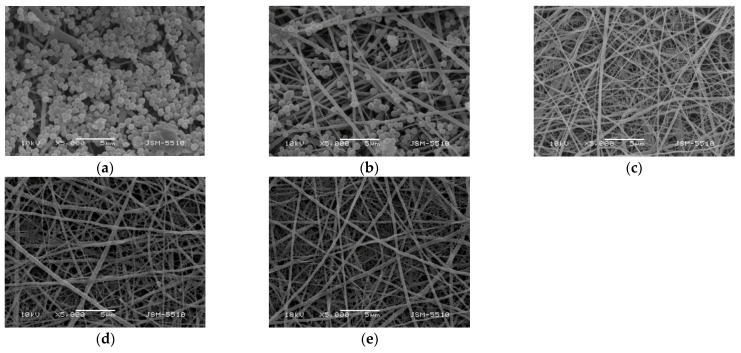
SEM micrographs of mats that have been incubated in *S. aureus* cell culture (10^7^ cells/mL) for 24 h at 37 °C, (**a**) PLA, (**b**) Ch/PLA, (**c**) Ch-8Q/PLA, (**d**) Cu^2+^ complex of Ch-8Q/PLA and (**e**) Fe^3+^ complex of Ch-8Q/PLA; magnification ×5000.

The adhesion of *S. aureus* cells on the surface of the fibrous materials was monitored by SEM. SEM micrographs of *S. aureus* cells adhered to the surface of the mats after contact with the *S. aureus* suspension for 24 h at 37 °C are presented in Figure 10. The *S. aureus* cells adhered very well to the surface of the hydrophobic PLA mat with a tendency to form a biofilm (Figure 10a). In the case of the Ch/PLA mats, the number of adhered bacterial cells decreased. The reduced adhesion of bacterial cells was most likely due to the presence of Ch, which had an antibacterial effect (Figure 10b).

The incorporation of Ch-8Q in the fibers, as well as the complex formation with Cu^2+^ and Fe^3+^, led to a complete suppression of the growth of *S. aureus* bacteria on the surface of the mats (Figure 10d,e). These results show that pathogenic bacteria were killed on contact with the mats, which was attributed to the high bactericidal activity of Ch-8Q incorporated in the mats and their Cu^2+^ and Fe^3+^ complexes. The obtained Ch-8Q-containing fibrous materials and their complexes might be suitable candidates as materials having a surface that can kill the pathogenic bacteria *S. aureus*.

### 3.8. Cytotoxicity of Fibrous Mats against HeLa and MCF-7 Cells and BALB/c 3T3 Fibroblasts

**Figure 11:**
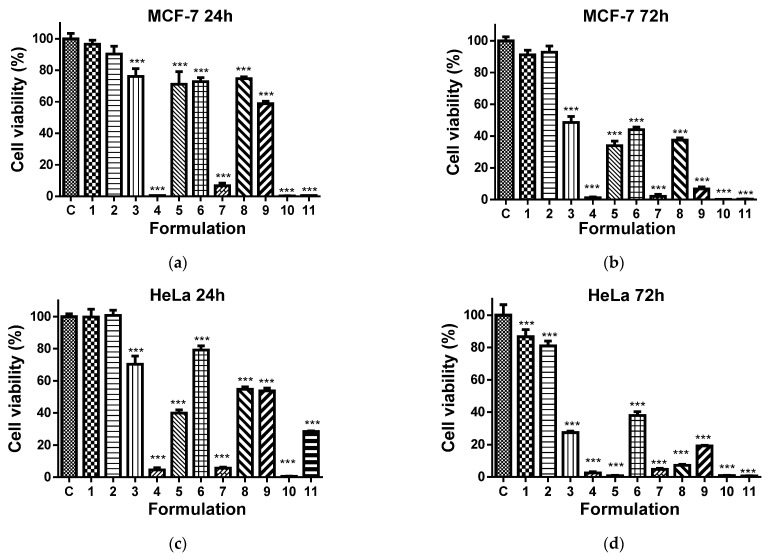

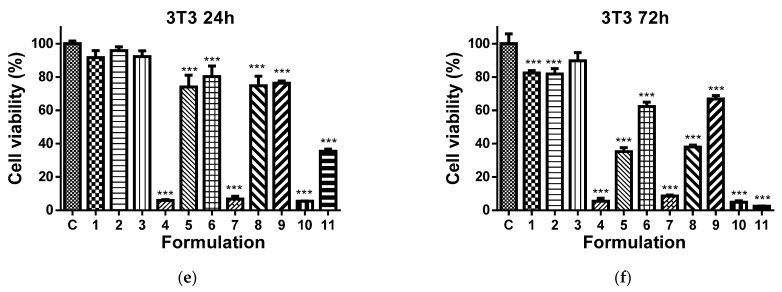
Effect of the different formulations on MCF-7 (**a**,**b**), HeLa (**c**,**d**), or BALB/c 3T3 (**e**,**f**) cells after 24 (**a**,**c**,**e**) and 72 h (**b**,**d**,**f**) of incubation. C—Untreated cells (control); 1—PLA mat; 2—Ch/PLA mat; 3—Ch-8Q/PLA mat; 4—Cu^2+^ complex of Ch-8Q/PLA mat; 5—Fe^3+^ complex of Ch-8Q/PLA mat; 6—aqueous solution of Jeff-8Q; 7—aqueous solution of Cu^2+^ complex of Jeff-8Q; 8—aqueous solution of Fe^3+^ complex of Jeff-8Q; 9—solution of 8QCHO; 10—solution of Cu^2+^ complex of 8QCHO and 11—solution of Fe^3+^ complex of 8QCHO. All 8Q-containing formulations were studied at a concentration of 8Q residues 60 µg/mL of culture medium. *** *p* < 0.001.

It has been reported that 8QCHO exerts good antiproliferative activity against various human cancer cell lines, such as Hs578t, SaoS2, K562, MDA231, SKHep1, T-47D and Hep3B [61]. In the present study, the viability of MCF-7 and HeLa cancer cells cultured in the presence of Ch-8Q/PLA fibrous materials and their complexes was evaluated by the MTT assay. The cytotoxic effect of these materials against non-cancer BALB/c 3T3 mouse fibroblast cells in vitro was also assessed. In this assay, 8QCHO and its Cu^2+^ (Fe^3+^) complexes, as well as Jeff-8Q and its Cu^2+^(Fe^3+^) complexes, were used as positive controls, and untreated MCF-7, HeLa, or BALB/c 3T3 cells were used as negative controls. The effect on cancer cell viability was less pronounced when they were treated with PLA and Ch/PLA mats (Figure 11a–d). In contrast, the viability of MCF-7 and HeLa cells treated with the Ch-8Q-containing fibrous materials and their complexes decreased significantly (Figure 11a–d). The observed antiproliferative effect increased on increasing the duration of the incubation period. The highest cytotoxicity of the Ch-8Q/PLA mats and their complexes was detected after 72 h of incubation (Figure 11b,d). The antiproliferative activity of these mats was more pronounced against HeLa cancer cells than MCF-7 cells. At the 72nd h of incubation, Cu^2+^ and Fe^3+^ complexes of Ch-8Q/PLA mats exhibited higher cytotoxicity (2.6 ± 1.8% and 0.8 ± 0.7% viable cells for Cu^2+^ and Fe^3+^ complexes of the mats, respectively) against HeLa cells compared to the Ch-8Q-containing mats (27.4 ± 2.1% viable cells). In the case of Cu^2+^ complexes of Ch-8Q/PLA mats, a stronger decrease in the proliferative activity of MCF-7 cancer cells (1.2 ± 1.3% viable cells) than that caused by Ch-8Q/PLA mats (48.6 ± 8.5% viable cells) and their Fe^3+^ complexes (34.0 ± 6.6% viable cells) was found. The percentage of viable HeLa and MCF-7 cells for the Ch-8Q-containing mats was close to that for free Jeff-8Q (approx. 37.9% viable HeLa cells and approx. 44.1% viable MCF-7 cells). About 4.8 ± 1.4 and 7.2 ± 1.8% of HeLa cells and about 2.1 ± 3.1 and 37.5 ± 3.7% of MCF-7 cells remained viable after 72h of incubation in the presence of Cu^2+^ and Fe^3+^ complexes of Jeff-8Q, respectively. These complexes had cytotoxicity close to that of the complexes of Ch-8Q/PLA mats. As seen in Figure 11b,d, 8QCHO and its complexes exhibited higher antiproliferative effects against both types of cancer cells than that of Ch-8Q-containing fibrous materials and its complexes. It should be noted that the Ch-8Q/PLA fibrous mats did not show any statistically significant antiproliferative activity against non-cancer BALB/c 3T3 cells—viable cells were 90.0 ± 12.2% after 72 h of incubation. PLA and Ch/PLA mats also exhibited low cytotoxicity against BALB/c 3T3 cells (Figure 11e,f). In the case of the complexes of Ch-8Q/PLA mats, the decrease in viability of BALB/c 3T3 cells was less pronounced compared to that for both types of cancer cells (Figure 11e,f). After 72 h of incubation, the percentage of viable BALB/c 3T3 cells was 35.3 ± 4.0% and 5.4 ± 3.7% for Fe^3+^ and Cu^2+^ complexes of the Ch-8Q/PLA mats, respectively. Therefore, Ch-8Q-containing mats and their complexes exerted high anticancer activity against HeLa and MCF-7 cells, while being less toxic against normal mouse BALB/c 3T3 fibroblasts.

### 3.9. Analysis of Cell Death by Staining Methods

**Figure 12:**
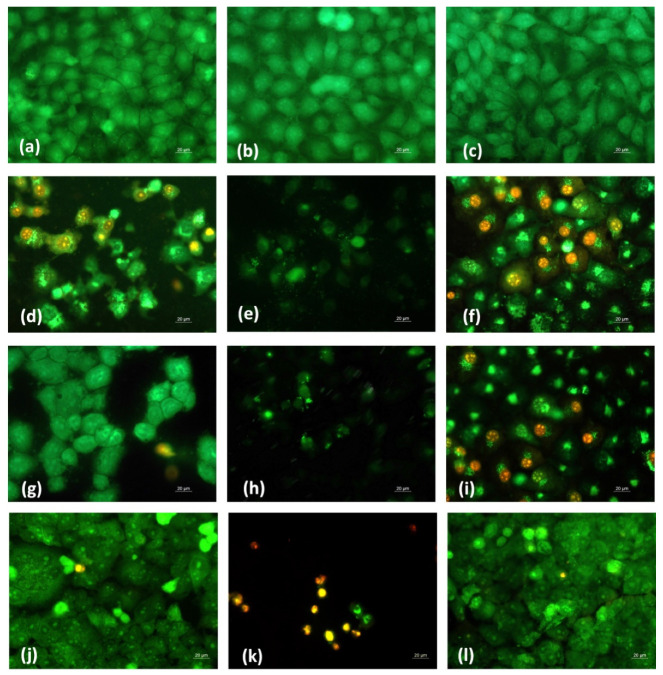
Fluorescence micrographs of AO and EtBr double-stained HeLa cancer cells incubated with different formulations for 24 h. Cells after incubation with (**a**) untreated HeLa cells, (**b**) PLA mat, (**c**) Ch/PLA mat, (**d**) Ch-8Q/PLA mat, (**e**) Cu^2+^ complex of Ch-8Q/PLA mat, (**f**) Fe^3+^ complex of Ch-8Q/PLA mat, (**g**) aqueous solution of Jeff-8Q, (**h**) aqueous solution of Cu^2+^ complex of Jeff-8Q, (**i**) aqueous solution of Fe^3+^ complex of Jeff-8Q, (**j**), solution of 8QCHO, (**k**) solution of Cu^2+^ complex of 8QCHO and (**l**) solution of Fe^3+^ complex of 8QCHO, scale bar = 20 µm. All 8Q-containing formulations were studied at a concentration of 8Q residues 60 µg/mL of culture medium.

In order to determine whether the antiproliferative effect of fibrous Ch-8Q-containing mats and their Cu^2+^ and Fe^3+^ complexes was related to the induction of apoptosis, a fluorescence assay was applied to detect cell death by intravital double staining with the fluorescent dyes AO and EtBr. The morphological features of HeLa and MCF-7 cancer cells cultured for 24 h in the presence of the various fibrous mats were studied. Untreated cancer cells (negative control) had homogeneous pale green nuclei and bright yellow-green nucleoli (Figure 12a and Supplementary Material Figure S4a).

No change was observed in the staining of the nuclei and cytoplasm in HeLa and MCF-7 cells after their treatment with PLA and Ch/PLA mats (Figure 12b,c and Supplementary Material Figure S4b,c). In these cases, the cell morphology remained normal. In contrast, when cells were cultured in the presence of the Ch-8Q/PLA mats and their complexes, cell rounding and cell shrinkage, cell membrane blebbing, cellular and nuclear volume reduction (pyknosis), condensation and aggregation of nuclear chromatin, the appearance of apoptotic bodies and nuclear fragmentation occurred (Figure 12d–f and Supplementary Material Figure S4d–f). These are typical morphological signs of early or late apoptosis. Similar morphological changes were observed when both types of cancer cells were placed in contact with free Jeff-8Q and its complexes or with free 8QCHO and its complexes (Figure 12g–l and Supplementary Material Figure S4g–l). The most significant morphological changes were detected in HeLa and MCF-7 cancer cells after their treatment with Cu^2+^ complexes of Ch-8Q/PLA mats, as well as with Cu^2+^ complexes of Jeff-8Q or 8QCHO (Figure 12e,h,k and Supplementary Material Figure S4e,h,k). A significant number of nuclei and cytoplasm of cells that were stained red-orange were observed, as well as a decrease in the number of cells and the presence of dead destructured cells with pyknotic nuclei (criteria for late apoptosis) (Figure 12e,h,k and Supplementary Material Figure S4e,h,k).

The morphological changes in the nuclei of HeLa and MCF-7 cancer cells were analyzed after staining the cells with DAPI. Control untreated HeLa and MCF-7 cells possessed intact nuclei, slightly oval, in shape, of approximately equal size, with smooth edges and uniformly distributed chromatin (Supplementary Material Figures S3a and S5a). The morphology of the nuclei of HeLa and MCF-7 cancer cells treated with PLA and Ch/PLA mats was close to that of the control (Supplementary Material Figures S3b,c and S5b,c). Cancer cells that had been in contact with the Ch-8Q/PLA mats and their complexes, with solutions of Jeff-8Q and its complexes, and with solutions of 8QCHO and its complexes were characterized by changes in the morphology of the nuclei, typical of apoptosis, such as chromatin condensation, nuclei pyknosis, nuclei fragmentation and an increase in the number of apoptotic bodies (Supplementary Material Figures S3d–l and S5d–l). The strongest damage to the nuclei of HeLa and MCF-7 cells was observed when they were treated with the Cu^2+^ complex of Ch-8Q/PLA mats or with Cu^2+^ complexes of Jeff-8Q and 8QCHO (Supplementary Material Figures S3e,h,k and S5e,h,k).

The obtained results were consistent with the data obtained from the MTT test and revealed that Cu^2+^ and Fe^3+^ complexes of Ch-8Q/PLA mats exerted a high antiproliferative effect against HeLa and MCF-7 cancer cells. Ch-8Q-containing mats displayed weaker cytotoxicity toward cancer cells. The observations from the performed fluorescence microscopy analyses showed that these mats induce the death of cancer cells via apoptosis.

When Balb/c 3T3 mouse fibroblasts were cultured in the presence of Cu^2+^ and Fe^3+^ complexes of Ch-8Q/PLA mats, morphological changes of the cells and nuclei that are characteristic of early and late apoptosis were detected (Supplementary Material Figures S6e,f and S7e,f). These changes were significantly greater in the case of Balb/c 3T3 cells treated with the Cu^2+^ complex of the Ch-8Q-containing mats (Supplementary Material Figures S6e and S7e). The results obtained from the fluorescence methods indicated that the Ch-8Q-containing mats did not exhibit toxicity against Balb/c 3T3 cells (Supplementary Material Figures S6d and S7d).

## 4. Conclusions

In the present study, the Schiff base derivative (Ch-8Q) of Ch and 8QCHO was synthesized and novel fibrous materials were successfully fabricated from Ch-8Q and PLA by one-pot electrospinning of their blend solution. Complexes of the mats were easily obtained by treating them with CuCl_2_ or FeCl_3_ solution. Based on ATR-FTIR, XPS and EPR spectroscopic analyses, it was concluded that Cu^2+^ and Fe^3+^ ions in both studied complexes of the Ch-8Q/PLA mats were preferably surrounded by 8Q-residue of Ch-8Q fibrous materials. The incorporation of Ch-8Q in the fibrous mats and complexation with Cu^2+^(Fe^3+^) imparted significant biocidal activity against *S. aureus* bacteria. These mats demonstrated the ability to kill all *S. aureus* bacterial cells within a contact time of 3h. Furthermore, in contrast to the Ch-containing mats, which only reduce the adhesion of pathogenic bacteria *S. aureus*, Ch-8Q-containing materials and their complexes inhibit bacterial adhesion. These fibrous mats exhibited high anticancer effects against human cervical HeLa and human breast MCF-7 carcinoma cell lines. Their in vitro anticancer activity depends on the incubation period. Fluorescence microscopy analyses indicated that the induction of apoptosis was one of the major mechanisms of the anticancer efficacy of the new materials. The cytotoxic effect of the mats was higher in cancer cells than in non-cancer BALB/c 3T3 mouse fibroblasts. Moreover, Ch-8Q/PLA mats displayed no cytotoxicity to the non-cancer cells. The obtained materials could find potential as wound dressing materials and in application in local treatment of cervical and breast cancer.

## Data Availability

The data presented in this study are available on request from the corresponding author.

## Supplementary Materials

The following supporting information can be downloaded at: https://www.mdpi.com/article/10.3390/polym14225002/s1, Figure S1: ATR-FTIR spectra of 8QCHO and Ch-8Q, Figure S2: XPS peak fittings for Ch-8Q/PLA mat, Figure S3: Fluorescence microscopic images of HeLa cancer cells stained with DAPI after treatment with PLA mat, Ch/PLA mat, Ch-8Q/PLA mat and its complexes, Jeff-8Q and its complexes and 8QCHO and its complexes, Figure S4: Fluorescence micrographs of AO and EtBr double-stained MCF-7 cancer cells incubated with different formulations for 24 h, Figure S5: Fluorescence microscopic images of MCF-7 cancer cells stained with DAPI after treatment with PLA mat, Ch/PLA mat, Ch-8Q/PLA mat and its complexes, Jeff-8Q and its complexes and 8QCHO and its complexes, Figure S6: Fluorescence micrographs of AO and EtBr double-stained BALB/c 3T3 cells incubated with different formulations for 24 h, Figure S7: Fluorescence microscopic images of BALB/c 3T3 cells stained with DAPI after treatment with PLA mat, Ch/PLA mat, Ch-8Q/PLA mat and its complexes, Jeff-8Q and its complexes and 8QCHO and its complexes.
