# Supplementary material for: Bio-Based Electrospun Fibers from Chitosan Schiff Base and Polylactide and Their Cu2+ and Fe3+ Complexes: Preparation and Antibacterial and Anticancer Activities
Source: Polymers (Basel). 2022 Nov 18;14(22):5002. doi: 10.3390/polym14225002 (PMC9696307; doi:10.3390/polym14225002)
Supplement: Supplementary file 1 [file polymers-14-05002-s001.zip › polymers-2021922-supplementary.pdf]

polymers

MDPI

Supplementary Materials

Preparation of the Cu^{2+}(Fe^{3+}) Complexes of Ch-8Q, of 8QCHO and of Jeff-*8Q*

The Cu^{2+} complex of Ch-8Q was prepared by adding ethanol solution of $CuCl_2$ (0.054 g, 0.4 mmol; 5 mL) to a suspension of Ch-8Q (0.112 g, 0.4 mmol) in ethanol (5 mL). The reaction mixture was heated at 40 °C for 48 h. The resulting dark green complex was filtered out, washed with ethanol, diethyl ether and dried under reduced pressure. Yield: 73%.

A similar procedure to that for the synthesis of the Cu^{2+} complex was used for the obtaining of Fe^{3+} complex of Ch-8Q. The complex that formed was collected by filtration, washed with ethanol, diethyl ether and dried under vacuum. Yield: 71%.

The Fe^{3+} complex of 8QCHO (molar ratio of 8Q and Fe^{3+} (1:1) was synthesized by mixing an ethanol solution of 8QCHO (0.013 g, 0.075 mmol; 10 mL) with 5 mL solution of $FeCl_3$ (0.0122 g, 0.075 mmo) in ethanol and then the mixture was shaken for 4h at 25 °C. For the preparation of Fe^{3+} complex of Jeff-8Q, a 5 mL ethanol solution of Jeff-8Q (0.06 g) was added to a solution of $FeCl_3$ (0.0061 g) in ethanol (5 mL) and the reaction mixture was stirred at room temperature for 4 h. The molar ratio of 8Q end groups of Jeff-8Q and Fe^{3+} was 1:1. The prepared ethanol solutions were cast on Petri dishes and dried under reduced pressure.

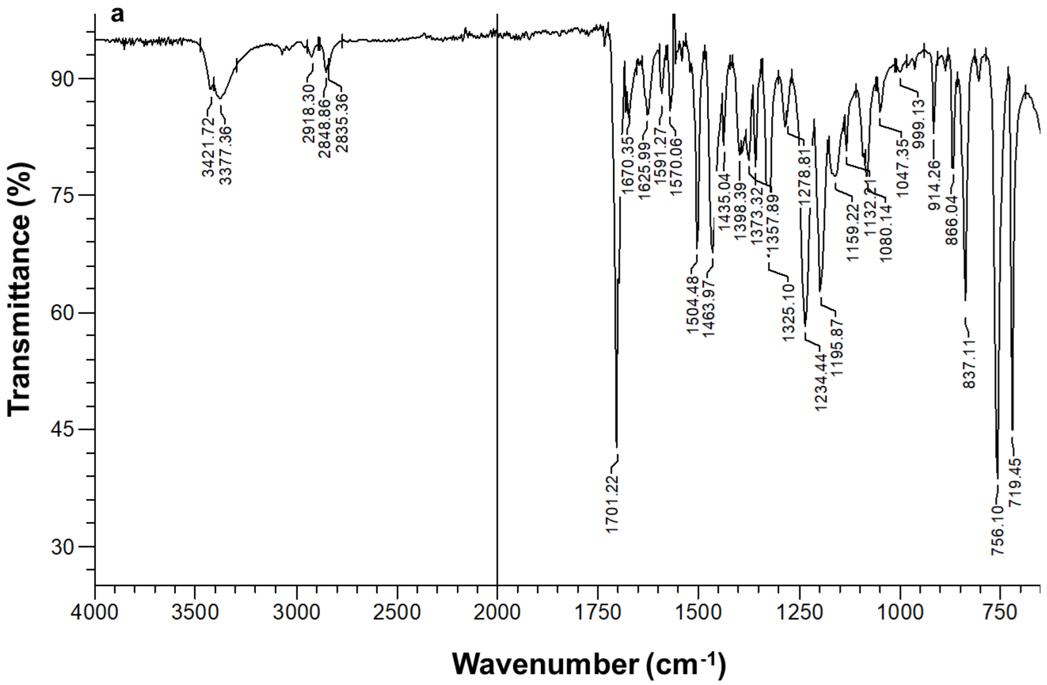

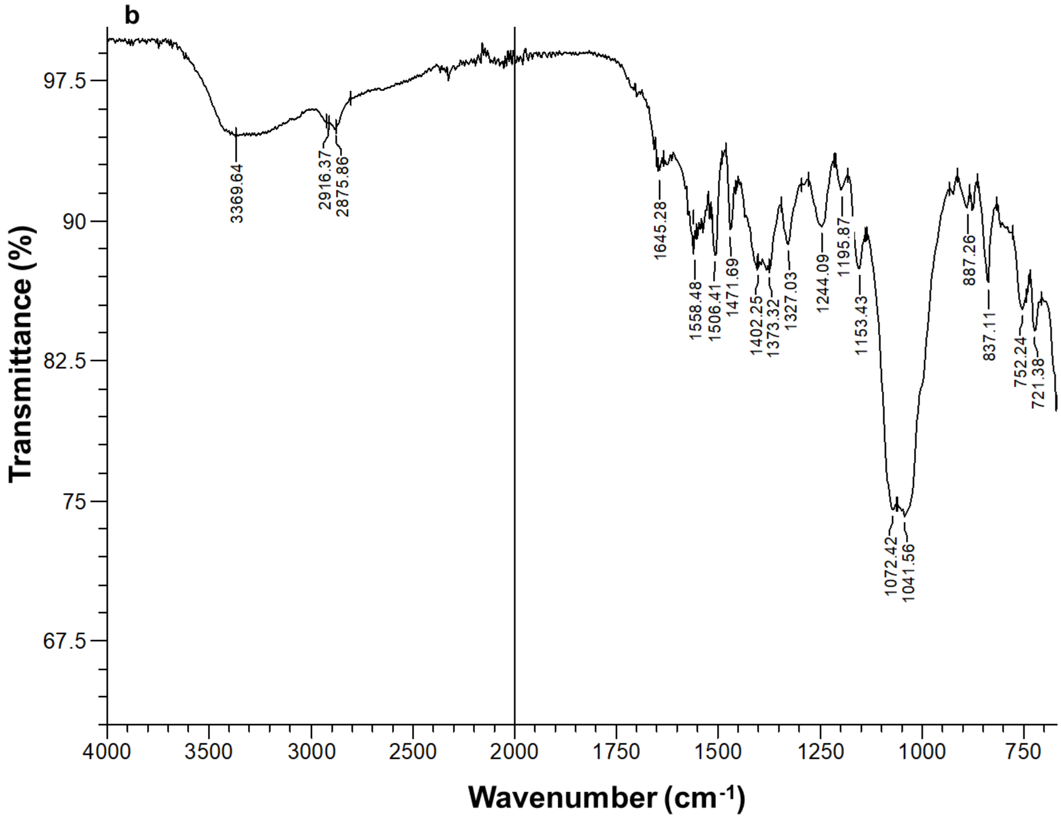

Figure S1. ATR-FTIR spectra of: (a) 8QCHO and (b) Ch-8Q.

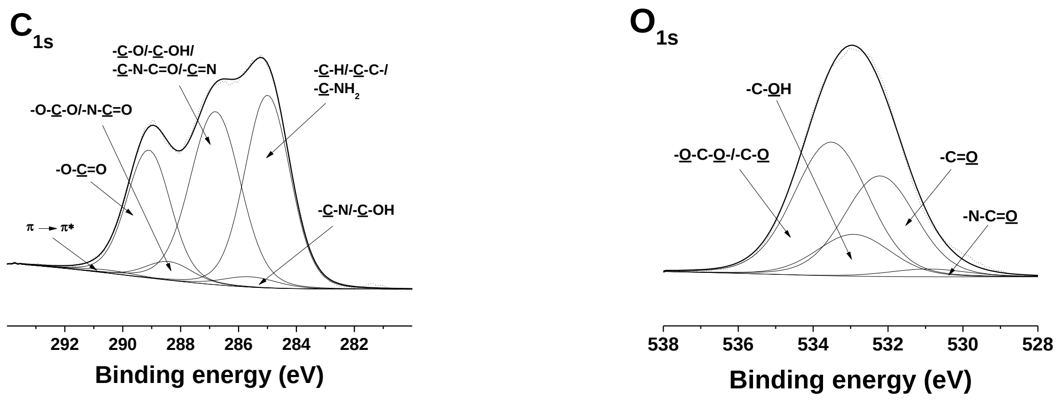

(**a**) (**b**)

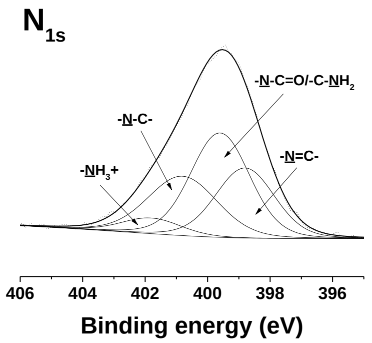

(**c**)

Figure S2. XPS peak fittings for Ch-8Q/PLA mat [C_{1s} (**a**), O_{1s} (**b**) and N_{1s} (**c**)].

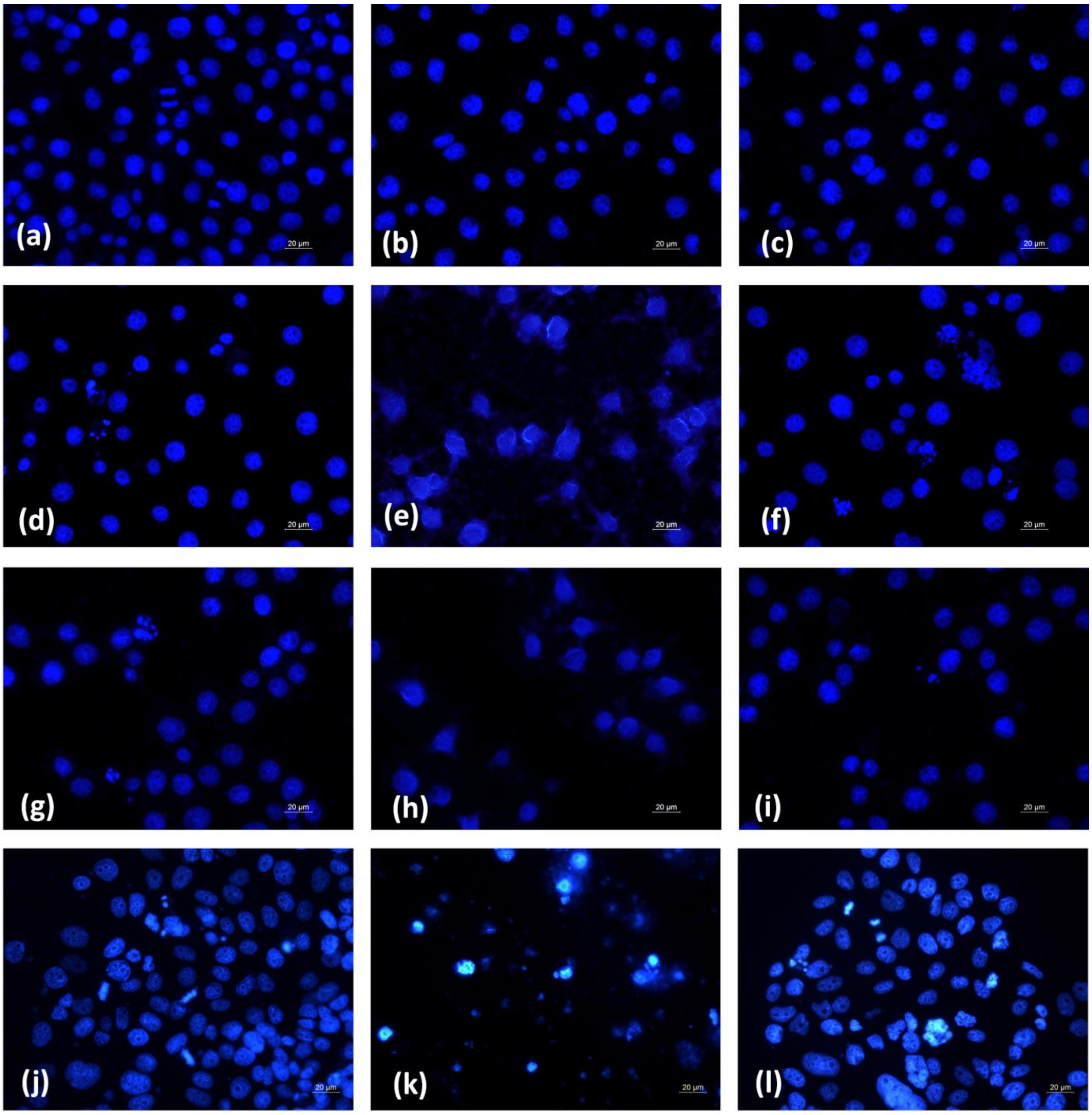

Figure S3. Fluorescence microscopic images of HeLa cancer cells stained with DAPI: (**a**) untreated HeLa cells (a control), (**b**) PLA mat, (**c**) Ch/PLA mat, (d) Ch-8Q/PLA mat, (**e**) Cu^{2+} complex of Ch-8Q/PLA mat, (**f**) Fe^{3+} complex of Ch-8Q/PLA mat, (**g**) aqueous solution of Jeff-8Q, (h) aqueous solution of Cu^{2+} complex of Jeff-8Q, (**i**) aqueous solution of Fe^{3+} complex of Jeff-8Q, (**j**), solution of 8QCHO, (k) solution of Cu^{2+} complex of 8QCHO, and (**l**) solution of Fe^{3+} complex of 8QCHO, scale bar = 20 µm. All 8Q-containing formulations were studied at concentration of 8Q residues 60 µg/ml of culture medium.

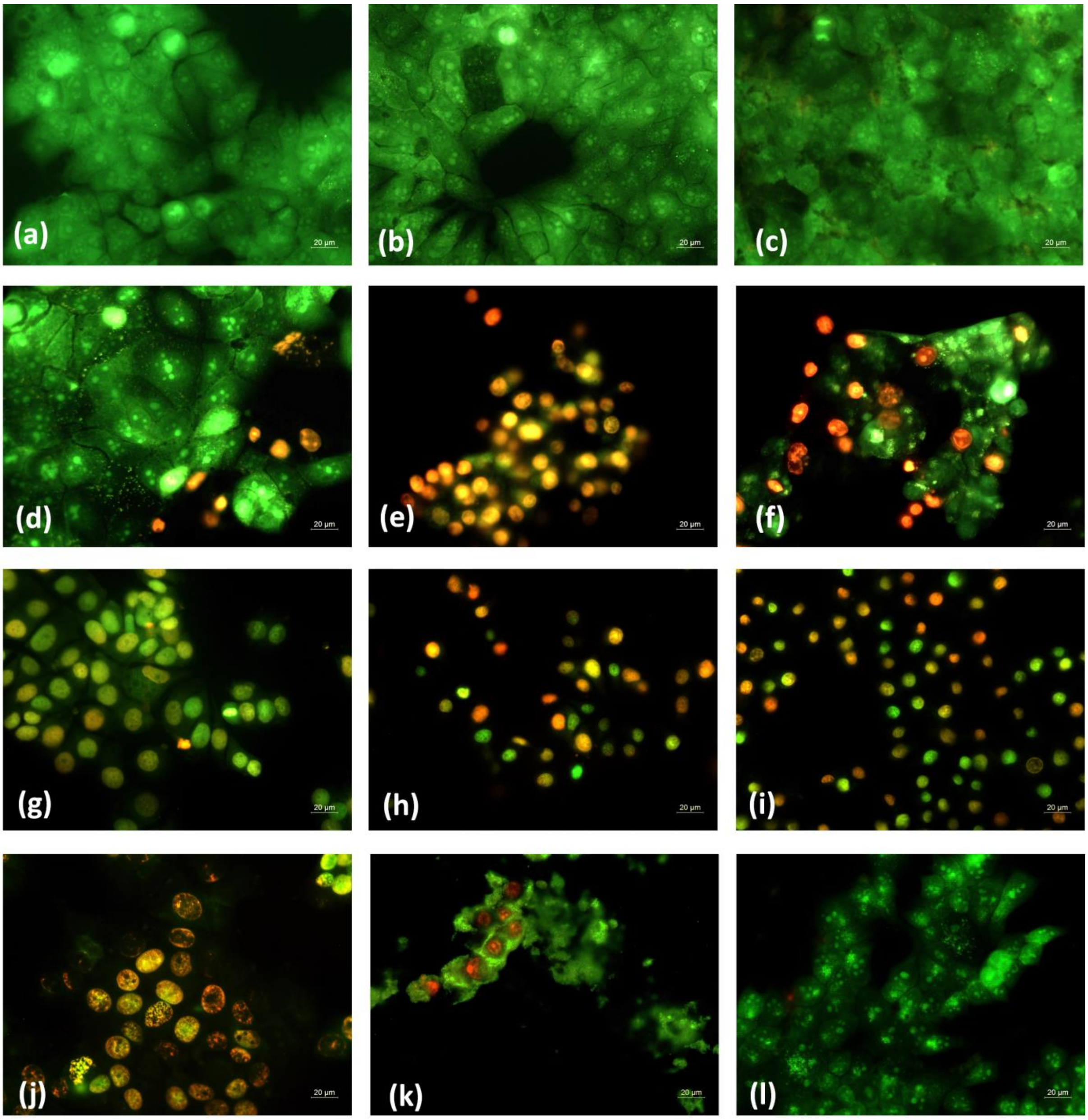

Figure S4. Fluorescence micrographs of AO and EtBr double-stained MCF-7 cancer cells incubated with different formulations for 24 h. Cells after incubation with: (**a**) untreated MCF-7 cells, (**b**) PLA mat, (**c**) Ch/PLA mat, (d) Ch-8Q/PLA mat, (e) Cu^{2+} complex of Ch-8Q/PLA mat, (**f**) Fe^{3+} complex of Ch-8Q/PLA mat, (**g**) aqueous solution of Jeff-8Q, (**h**) aqueous solution of Cu^{2+} complex of Jeff-8Q, (**i**) aqueous solution of Fe^{3+} complex of Jeff-8Q, (**j**), solution of 8QCHO, (**k**) solution of Cu^{2+} complex of 8QCHO, and (**l**) solution of Fe^{3+} complex of 8QCHO, scale bar = 20 μm. All 8Q-containing formulations were studied at concentration of 8Q residues 60 μg/ml of culture medium.

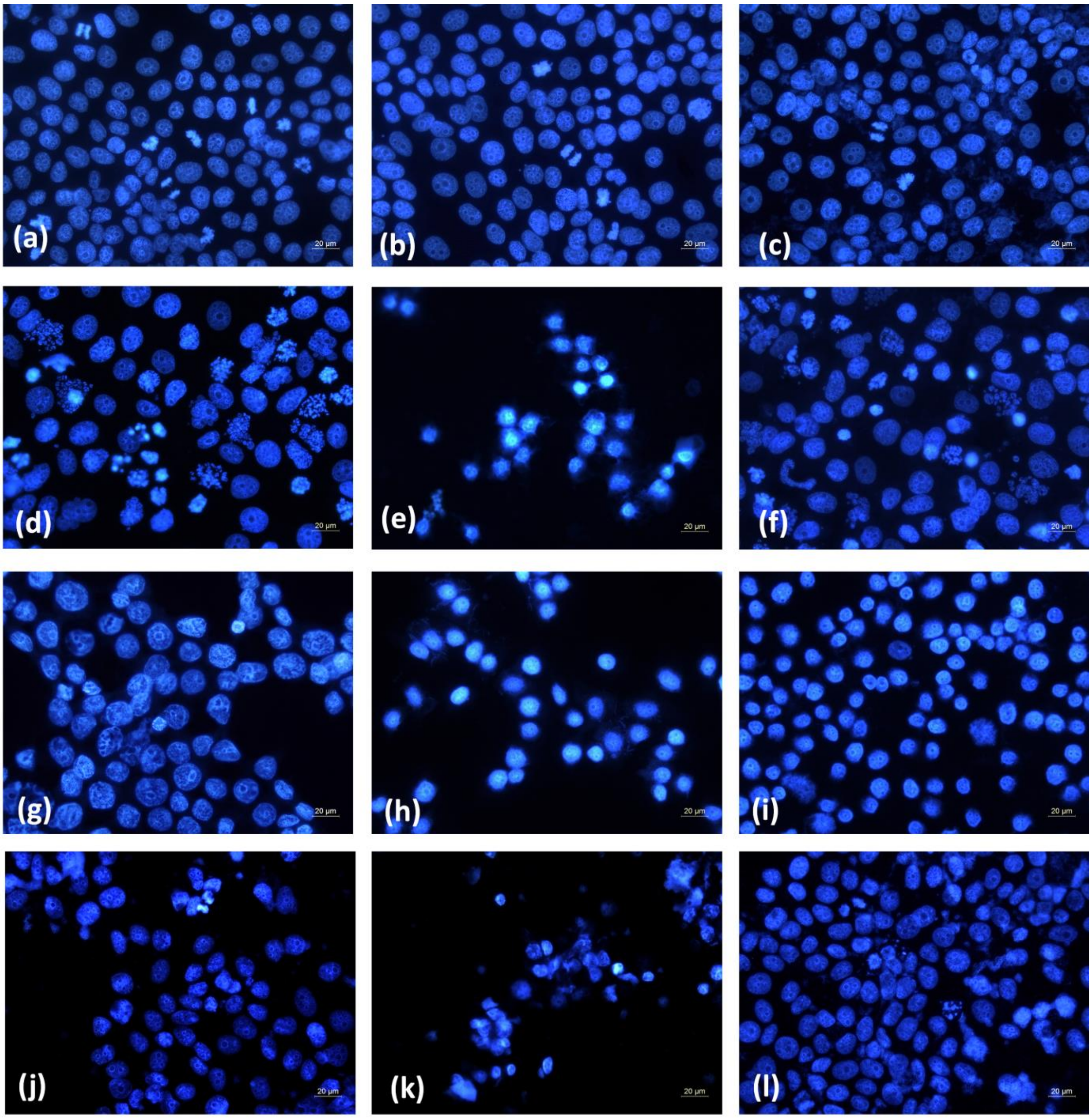

Figure S5. Fluorescence microscopic images of MCF-7 cancer cells stained with DAPI: (**a**) untreated MCF-7 cells (a control), (b) PLA mat, (**c**) Ch/PLA mat, (**d**) Ch-8Q/PLA mat, (**e**) Cu^{2+} complex of Ch-8Q/PLA mat, (**f**) Fe^{3+} complex of Ch-8Q/PLA mat, (g) aqueous solution of Jeff-8Q, (**h**) aqueous solution of Cu^{2+} complex of Jeff-8Q, (**i**) aqueous solution of Fe^{3+} complex of Jeff-8Q, (**j**), solution of 8QCHO, (**k**) solution of Cu^{2+} complex of 8QCHO, and (**l**) solution of Fe^{3+} complex of 8QCHO, scale bar = 20 μm. All 8Q-containing formulations were studied at concentration of 8Q residues 60 μg/ml of culture medium.

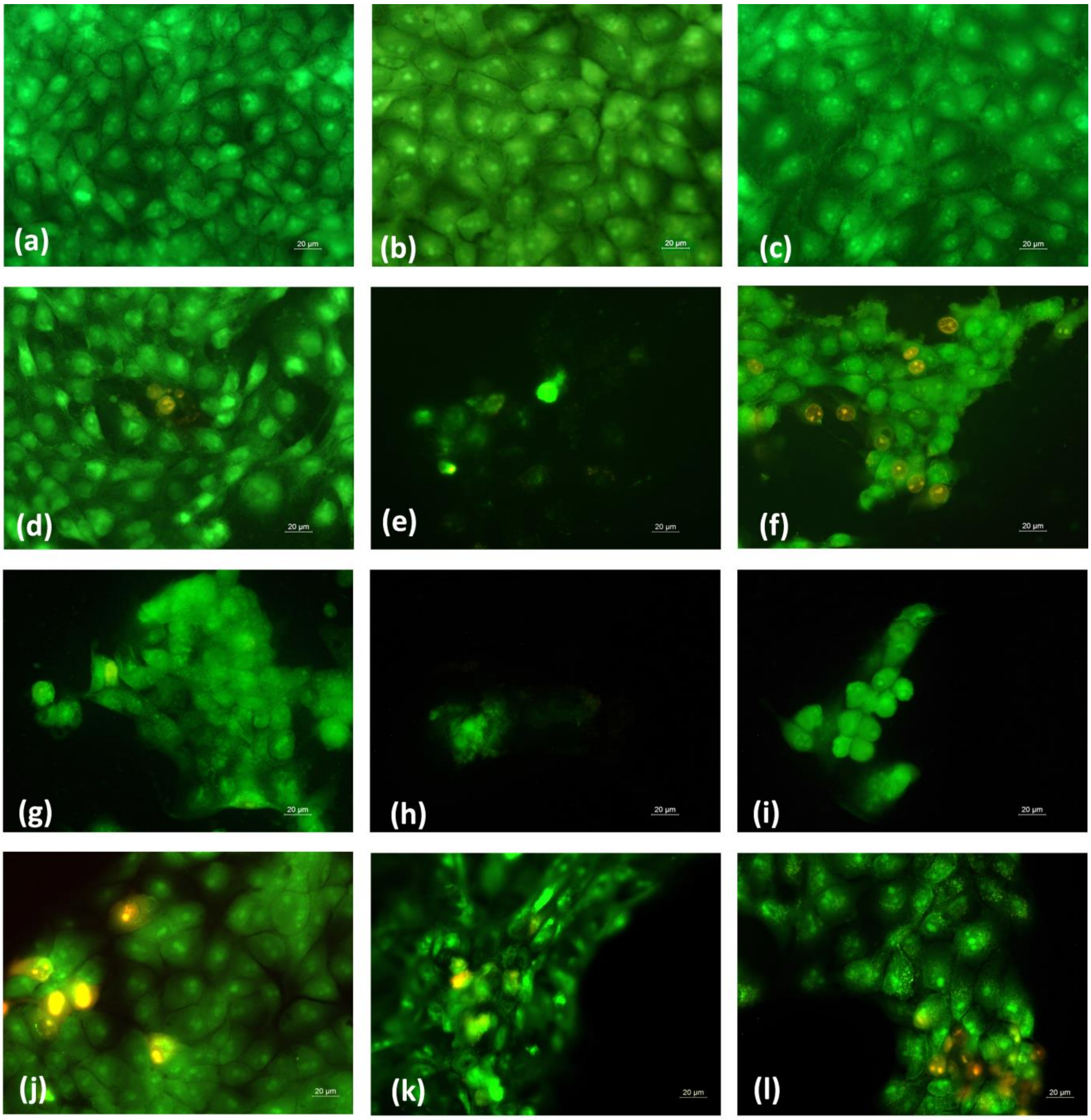

Figure S6. Fluorescence micrographs of AO and EtBr double-stained BALB/c 3T3 cells incubated with different formulations for 24 h. Cells after incubation with: (a) untreated BALB/c 3T3 cells, (b) PLA mat, (c) Ch/PLA mat, (d) Ch-8Q/PLA mat, (e) Cu^{2+} complex of Ch-8Q/PLA mat, (f) Fe^{3+} complex of Ch-8Q/PLA mat, (g) aqueous solution of Jeff-8Q, (h) aqueous solution of Cu^{2+} complex of Jeff-8Q, (i) aqueous solution of Fe^{3+} complex of Jeff-8Q, (j), solution of 8QCHO, (k) solution of Cu^{2+} complex of 8QCHO, and (l) solution of Fe^{3+} complex of 8QCHO, scale bar = 20 μm. All 8Q-containing formulations were studied at concentration of 8Q residues 60 μg/ml of culture medium.

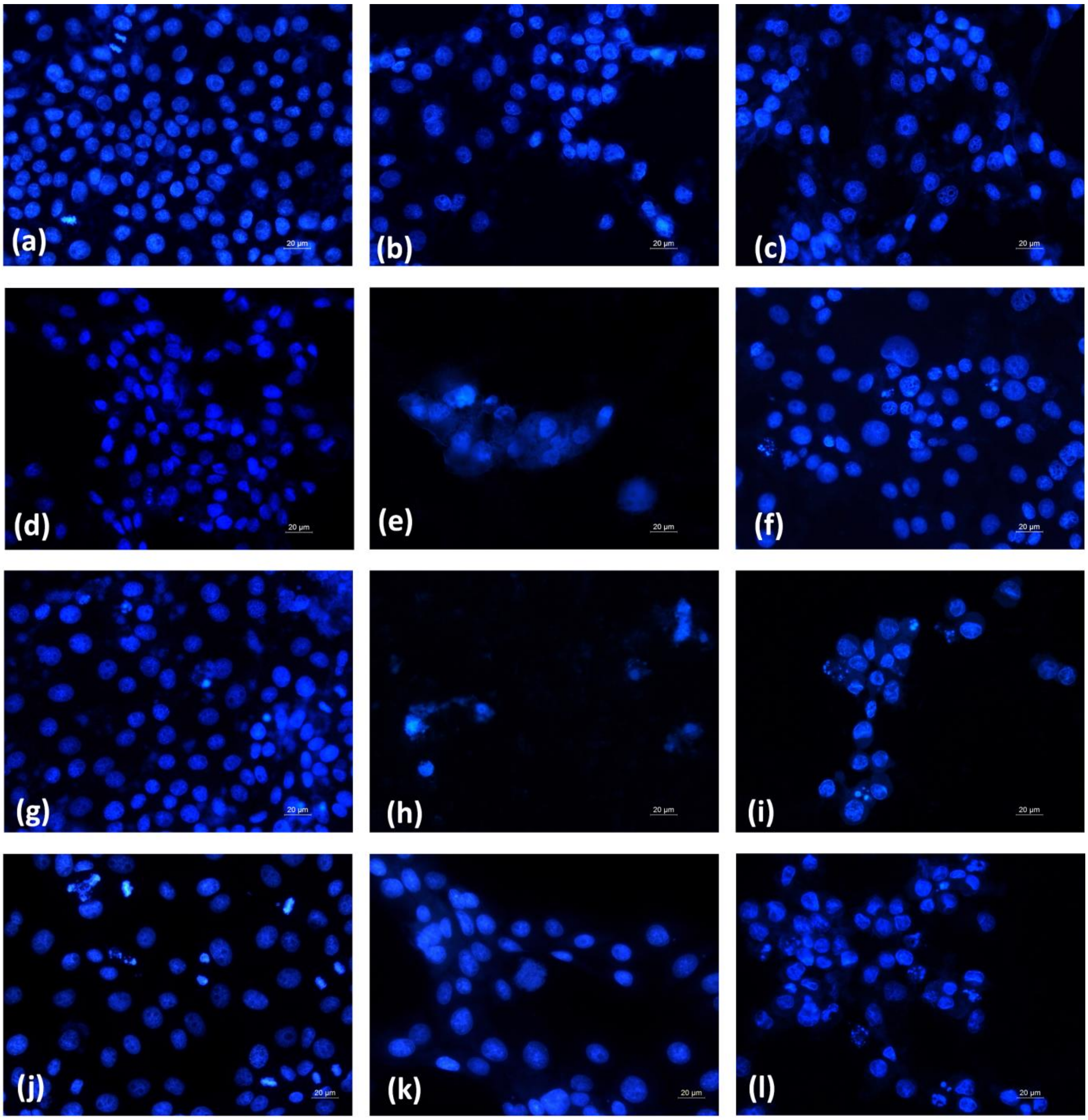

Figure S7. Fluorescence microscopic images of BALB/c 3T3 cells stained with DAPI: (a) untreated BALB/c 3T3 cells (a control), (b) PLA mat, (c) Ch/PLA mat, (d) Ch-8Q/PLA mat, (e) Cu^{2+} complex of Ch-8Q/PLA mat, (f) Fe^{3+} complex of Ch-8Q/PLA mat, (g) aqueous solution of Jeff-8Q, (h) aqueous solution of Cu^{2+} complex of Jeff-8Q, (i) aqueous solution of Fe^{3+} complex of Jeff-8Q, (j), solution of 8QCHO, (k) solution of Cu^{2+} complex of 8QCHO, and (l) solution of Fe^{3+} complex of 8QCHO, scale bar = 20 μm. All 8Q-containing formulations were studied at concentration of 8Q residues 60 μg/ml of culture medium.
